# Pedigree-based Bayesian modelling of radiocarbon dates

**DOI:** 10.1371/journal.pone.0270374

**Published:** 2022-06-30

**Authors:** Ken Massy, Ronny Friedrich, Alissa Mittnik, Philipp W. Stockhammer

**Affiliations:** 1 Institute for Pre- and Protohistoric Archaeology and Archaeology of the Roman Provinces, Ludwig-Maximilians-University Munich, Munich, Germany; 2 Curt Engelhorn Center for Archaeometry, Mannheim, Germany; 3 Department of Human Evolutionary Biology, Harvard University, Cambridge, Massachusetts, United States of America; 4 Department of Genetics, Harvard Medical School, Boston, Massachusetts, United States of America; 5 Max Planck Harvard Research Center for the Archaeoscience of the Ancient Mediterranean, Max Planck Institute for Evolutionary Anthropology, Leipzig, Germany; Univiersity of Vienna, AUSTRIA

## Abstract

Within the last decade, archaeogenetic analysis has revolutionized archaeological research and enabled novel insights into mobility, relatedness and health of past societies. Now, it is possible to develop these results further and integrate archaeogenetic insights into biological relatedness with radiocarbon dates as means of chronologically sequenced information. In our article, we demonstrate the potential of combining relative chronological information with absolute radiocarbon dates by Bayesian interpretation in order to improve age determinations. Using artificial pedigrees with four sets of simulated radiocarbon dates we show that the combination of relationship information with radiocarbon dates improves the age determination in many cases at least between 20 to 50%. Calibrated age ranges are more constrained than simply calibrating radiocarbon ages independently from each other. Thereby, the precision of modelled ages depends on the precision of the single radiocarbon dates, the number of modelled generations, the shape of the calibration curve and the availability of samples that can be precisely fixed in time due to specific patterns in the calibration curve (“anchor points”). Ambiguous calibrated radiocarbon dates, which are caused by inversions of the calibration curve, can be partly or almost entirely resolved through Bayesian modelling based upon information from pedigrees. Finally, we discuss selected case studies of biological pedigrees achieved for Early Bronze Age Southern Germany by recent archaeogenetic analysis, whereby the sites and pedigrees differ with regard to the quality of information, which can be used for a Bayesian model of the radiocarbon dates. In accordance with the abstract models, radiocarbon dates can again be better constrained and are therefore more applicable for archaeological interpretation and chronological placement of the dated individuals.

## Introduction

Radiocarbon dating, developed by Willard F. Libby in the 1940s, led to a revolution in our understanding of our past [1]. Previously, mainly relative chronological information was available to archaeologists. With the introduction of radiocarbon dating, it became possible to scientifically date events or objects absolutely and assign them to calendric years for the first time. Conventional radiocarbon dates are only an approximation of actual calendar dates and need to be converted to calendric dates by calibrating them against radiocarbon archives dated independently (e.g., dendrochronology). Those calibration data are available through the work of the IntCal working group [2] and can be used freely.

Since past atmospheric radiocarbon content varied over time, one high-precision radiocarbon measurement can result in multiple calibrated calendric date ranges, resulting in much larger uncertainties over the initial radiocarbon result. While more precise radiocarbon dates do improve the uncertainty of the calibrated dates and result in narrower age ranges, the nature of the atmospheric ^14^C fluctuations will always limit the calibrated uncertainty.

Bayesian modelling of radiocarbon data [3,4] has shown great success for analysing absolute chronological sequences. While radiocarbon dates alone, due to their absolute nature, are important, the combination of absolute and relative chronological information can yield much better constrained age information. However, additional information is needed, e.g. stratigraphic information that prescribes a chronological order of events or assumed anteriority and/or posteriority of archaeological events. Generally speaking, the more chronological information that is available, the more precise the overall outcome of the Bayesian model will be.

So far, such specifications in archaeological contexts are only available via physical stratigraphic contexts (see [5]), as for example occupation and destruction layers in tell settlements or sedimentation (c.f. [6]). However, one of the main sources for (chrono-)archaeological research are still cemeteries and their burials. Unfortunately, graves in cemeteries, from which most chronologically relevant type artefacts stem, rarely provide us with stratigraphic information because of the lack of grave intersections or other indications of their relative chronology (e.g., colluvial deposits or structural remains of buildings). Therefore, finding new ways to refine absolute chronological information, respectively constraining the age ranges of the calibrated dates gathered from skeletal material of graves, is imperative. New research in ancient DNA (e.g. [7–14]) and the resulting possibility to reconstruct pedigrees enable us to add chronological and sequential information to radiocarbon dates of those individuals. Those cornerstones of information can be used to set up a timeline of events, in our case the estimated times of death, which are the basis for Bayesian modelling of radiocarbon dates gained from each individual within the pedigree.

Mittnik et al. 2019 [11] has demonstrated for the first time that using pedigree information can place better constraints on calibrated ^1^⁴C ages, thus reducing the calibration ranges by informing the calibration procedure about the chronological order of the generations. Sedig et al. 2021 [15] introduced the concept of maximum date-of-death (DoD) separation. DoD’s of subsequent generations can only be separated by a maximum number of years, therefore placing additional constraints on calibrated ^14^C ages. In the following, the term generation is used according to the linguistic usage in ancient genome research describing the degree of relatedness and the position of individuals in a pedigree.

Based on these first attempts, we present a next step of Bayesian modelling of biological pedigrees. As a basis, we chose the already published genome-wide data of 104 individuals mainly of the Bell Beaker period and Early Bronze Age from the Lech Valley south of today’s city of Augsburg, Bavaria, which had already been used by us for testing the first steps in the Bayesian modelling of pedigrees. The complexity of this dataset, with its reconstruction of five pedigrees yielding up to five generations [11], presents the best possible basis to demonstrate the use of pedigree data as chronological information aiding radiocarbon dates in Bayesian models. The potential of the method is explored with simulated radiocarbon and pedigree data located in varying sections of the calibration curve and in real case studies (for a map of the discussed sites, see [11]). Different parameters, priors and variables will be discussed, as well as their consequences. Taking into account that ancient DNA and the resulting reconstruction of pedigrees will play an increasing role in upcoming archaeological and genetic studies, this paper serves as a first guideline.

## Materials and methods

### Preconditions

The general idea of the paper is to use the pedigrees provided by biological kinship analyses of ancient DNA to prescribe a chronological order to the year-of-death (YoD) of the individuals and to their ^14^C dates. This timeline is then used for statistical analyses (Bayesian modelling) of the ^14^C dates to refine and constrain the dating ranges of each single radiocarbon measurement, as well as for the chronological placement of the family tree. Therefore, three major preconditions and requirements must be met. First of all, a pedigree with at least two generations (parent[s] and child[ren]) is necessary to make statements about their relative chronological relation to each other. All individuals which can be placed within the pedigree must have been radiocarbon dated to increase the information density in the model. To avoid as many unknowns/uncertainties from the outset and to refine the overall outcome of the statistical models, the anthropologically determined age-at-death (AaD) of the individuals is of great advantage in quantifying the difference in calendric years between the years-of-death (YoDs) of each individual. In addition, we only used radiocarbon dates from uncremated skeletal remains to exclude any taphonomic issues associated with charcoals or other organic remains from the filling of the grave pit.

However, one main prior assumption has to be made–the reproductive age is set at 20 years of age for all simulations, determining the difference of the year-of-birth (YoB) from one generation-level to the next, which is plausible as a mean value for pre-industrial societies. This is necessary to calculate the generation gap/intervals used in the simulations, but we are well aware that it is rather a reproductive time span in reality [16,17]. However, the generation interval of YoBs of 20 years is also based upon observations of the mean values of the AaD of individuals within double burials yielding a mother and a child (c.f. Early Bronze Age references similar to the case studies presented in this paper: [18,19]. On the basis of different strands of archaeological and scientific evidence, Rebay-Salisbury and colleagues could determine the very start of the reproductive age of Bronze Age women at around 14–15 years of age, and at the other end of the reproductive time span, it seems plausible for 35- or 40-year-old women to have given birth [16]. For male ancestors, the age at which they became fathers is unknown and is therefore set at the same age as for women. In cases of more than one detected descendant buried in different graves than their parents, it is unfortunately not possible to decide on an archaeological basis which one was born first or how many years lie between the dates of births of siblings. Only graves containing two or more siblings buried (or who died) at the same time (e.g., three siblings in AITI_77: [11]) can give information about the individuals’ chronological order, meaning their YoB. Generally speaking, a sibling age gap from one to several years is plausible [16]; extreme cases with a YoD-difference of about 100 or more is possible [15], but very unlikely for past societies. When setting up pedigree-based Bayesian models for real cases, site- and case-specific information can lead to a modified reproductive age used for modelling, optimally for each of the persons of the family tree individually.

### ^14^C analysis

All measured radiocarbon dates used for the case studies (POST, OBKR and AITI) originate from cemeteries in the Lech Valley with almost exclusively inhumations and were part of the multidisciplinary WIN-project: “Times of Upheaval: Changes of Society and Landscape at the Beginning of the Bronze Age” [11,20–22]. Collagen was extracted from bone samples using a modified Longin-extraction method [23,24] followed by ultra-filtration and freeze-drying. The extracted collagen was combusted in an elemental analyzer (EA) to CO_2_, which was then reduced to graphite. The ^14^C analysis was performed at CEZA (Curt-Engelhorn-Center Archaeometry) using a MICADAS-type AMS system [25]. In the Elemental Analyzer, the C:N ratio can be determined. For all samples, the C:N ratio was in the accepted range of well-preserved bone (2.9–3.6) [20,26,27]. Based upon the analysis of stable carbon and nitrogen isotope ratios (δ^13^C and δ^15^N) of bone collagen of samples within the data set, no evidence of a reservoir effect caused by dietary habits was detected which could have influenced the results of radiocarbon dating [20]. Data evaluation and calculation of ^14^C ages was done using the software BATS [28]. OxCal 4.3 was used for calibration and Bayesian modelling by applying the calibration dataset IntCal13 [29], because IntCal20 [2] was only available at the end of the writing process of this paper. Nonetheless, the methods and results presented are valid for every calibration curve dataset. A random double check with IntCal13 and IntCal20 of single calibrated dates as well as Bayesian models used in this paper produced results which did not differ by more than 4 calendric years, as the sections of the calibration curve examined stayed almost entirely unchanged within the two versions. If not stated otherwise, all dating ranges of calibrated dates mentioned in the text represent 2-sigma probabilities (95,4%). All 2-sigma dating ranges in graphs, tables and time span calculations are based upon the entire length of the probability ranges, regardless of the gaps that occur due to peaks and wiggles of the calibration curve: e.g., 2550 ± 25 BP with its calibrated dates of 801–747 (64,4%), 685–666 (8,5%) and 642–554 (22,6%) cal BC is shown as 801–554 cal BC in sum.

The ^14^C age of the dated bone collagen does not reflect the time of death (ToD) but rather the time when carbon was incorporated into the bone material (human bone collagen offset: HBCO) [30]. The anthropological AaD information of the individuals within the pedigrees of the case studies enables us to include the HBCO correction (0–25 years offset) into the Bayesian model by subtracting the HBCO from the conventional ^14^C age before analysing them with statistical methods [31]. Both the intergenerational gap and HBCO correction can be incorporated in the model as preconditions before running the statistical process. Note that HBCO correction is not implied in the four simulations (plateau, steep, mixed and anchor point), because the radiocarbon ages equal the given/prescribed YoDs already.

### DNA and pedigree determination

To demonstrate our Bayesian modelling approach, we use the pedigrees as reported in Mittnik et al. 2019 [11] and summarize the methods to arrive at the pedigrees here. In the case study for Haunstetten—Postillionstraße (POST) we model both alternative pedigrees shown in the original paper (ref). Alternative pedigrees might be possible in the other case studies but are not analysed or discussed here. DNA extraction, preparation of immortalized DNA libraries, targeted capture for around 1.2 million single nucleotide polymorphisms (SNPs), which are informative about genetic ancestry [32], and sequencing were carried out as described in Mittnik et al. 2019 [11]. Relatedness between pairs of individuals was estimated on the basis of overlapping SNPs in the set of targeted SNPs, with 10,000 SNP positions covered in both individuals as a lower threshold using the software READ [33] to calculate *P*_*0*_, the average pairwise mismatch rate between individuals, and the software *lcMLkin* [34] to calculate the coefficient of relatedness *r*. Both of these measures determine the degree of relatedness. Additionally, *lcMLkin* estimates the Cottermann coefficients *k*_*0*_, *k*_*1*_ and *k*_*2*_, the probability that zero, one or two alleles, respectively, are shared, identical-by-descent, between two individuals. For example, *k*_*0*_ is expected to be 0 in parent-offspring pairs and 0.25 in full siblings and thus serves to separate these two types of first-degree relationships. Additional lines of evidence were used to reconstruct family trees: identical mitochondrial haplotypes indicate maternal kinship, and, while not as well resolved, the same Y-chromosome haplogroups could indicate paternal kinship. We can also draw on the AaD, since sub-adult individuals are unlikely to be parents (cf. pedigree of POST individuals 131 and 137).

### Simulation and model setups

A large set of models were created using simulated ^14^C ages in order to test the general applicability of combining pedigree information with ^14^C ages. Those models reflect important characteristics that may affect the accuracy and precision of calibrated ^14^C dates—the shape of the calibration curve, the number of generations comprising the model and certain model parameters such as knowledge about the gap between the generations and the HBCO correction. The software OxCal 4.3.2 [35] with the calibration dataset IntCal13 [29] was used for modelling and calibration.

**Fig 1:**
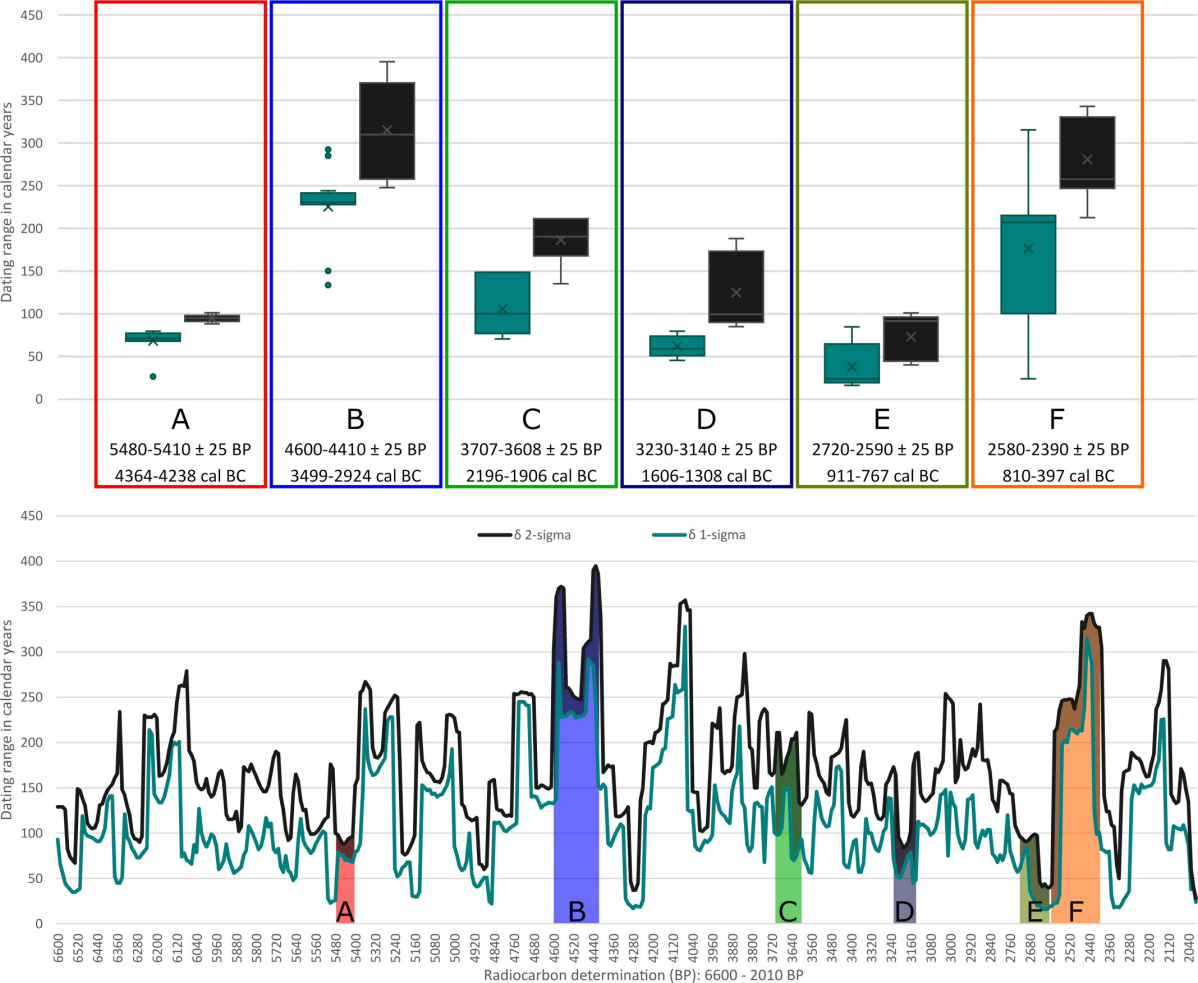
Ranges of calibrated radiocarbon dates from 6600–2021 BP. The compilation of calibrated radiocarbon dates (using the IntCal13 dataset) with regular intervals of 5 years (ca. 5600–1 cal BC: The Early Neolithic to the beginning of the Common Era in Central Europe) and with an uncertainty of +/- 25 years represent a typical uncertainty of modern AMS-dating laboratories. Representative sections of the calibration curve are chosen (A-F) to illustrate variations in their overall dating ranges (bottom panel) and differences between their 1- and 2-sigma ranges (Whisker-Box-plots in the upper panel). A: Steep sections of the calibration curve (5480–5410 BP); B: Extensive plateau section in the Neolithic period (4600–4410 BP; cf. Meadows et al. 2020 [5]); C: Early Bronze Age mixed section corresponding to our case studies (3700–3600 BP); D: Middle Bronze Age section with significant differences between 1- and 2-sigma ranges (3230–3140 BP); E: Short steep section of the calibration curve before the Hallstatt plateau (2720–2590 BP); F: Hallstatt plateau with two distinct sub-plateaus (2580–2390 BP).

Atmospheric ^14^C fluctuations, visible in the ups and downs of the calibration curve, result in periods with wider and narrower calibrated date ranges. While smaller uncertainties of the ^14^C ages are also reflected in narrower calibrated date ranges (see S1 Fig), the shape of the calibration curve largely determines the extent of the calibrated ranges (Fig 1).

In general, three types of simulations reflecting common shapes of the calibration curve as seen in various archaeological records were set up. Models identified as: (**Plateau**; Fig 1F) a plateau-rich calibration curve (Hallstatt plateau) between around 770–400 cal BC (^14^C ages around 2500 BP), (**Steep**; comparable to Fi. 1A) a calibration curve with steep sections at 7600–7500 cal BC (^14^C ages around 8600 BP) and (**Mixed**; Fig 1C) a “normal” section of the calibration curve at 2000–1800 cal BC (^14^C ages around 3600 BP) without any wide plateaus or steep sections.

**Fig 2:**
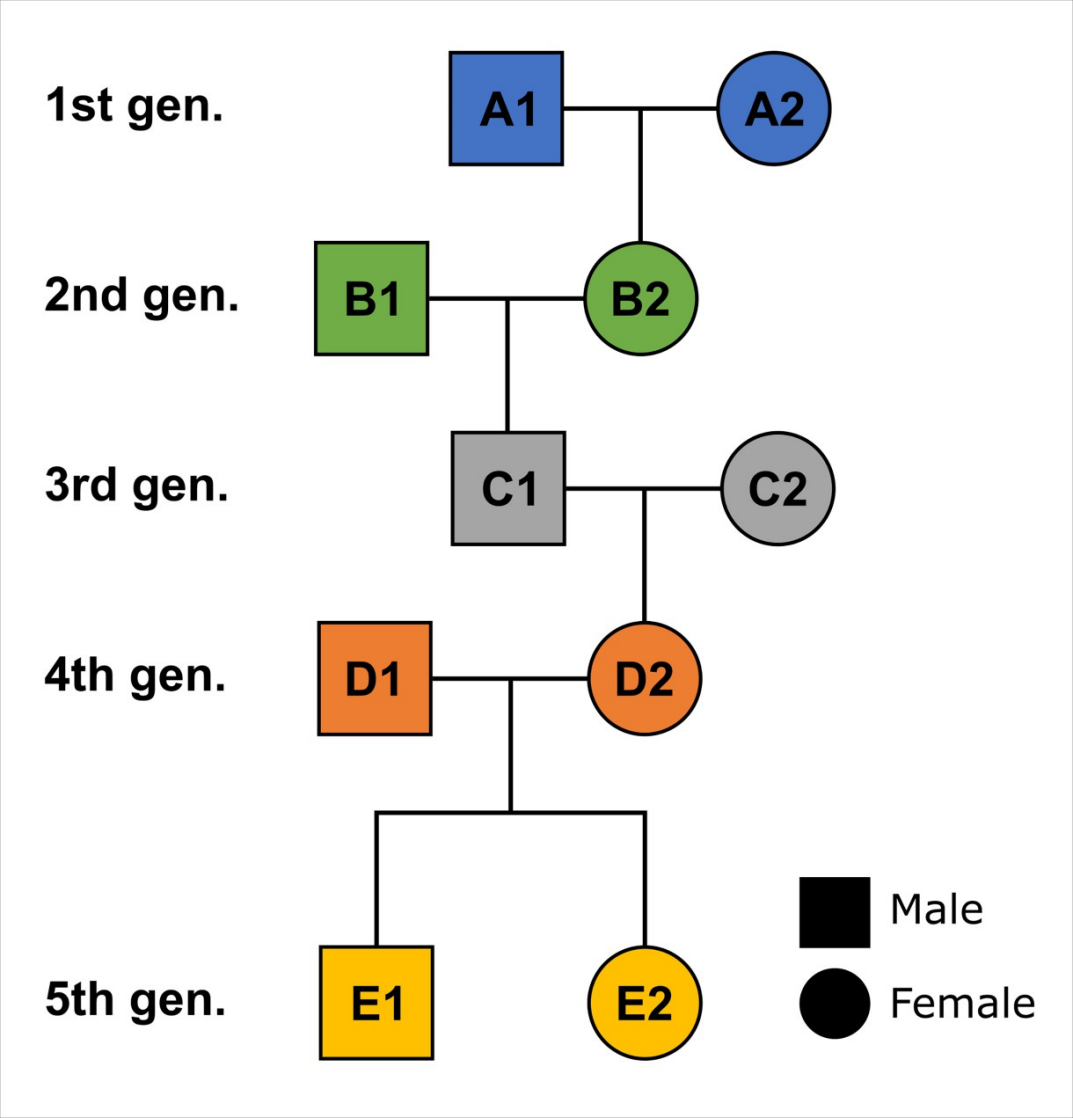
Simplified single-strand pedigree consisting of five generations used for the simulations.

**Fig 3:**
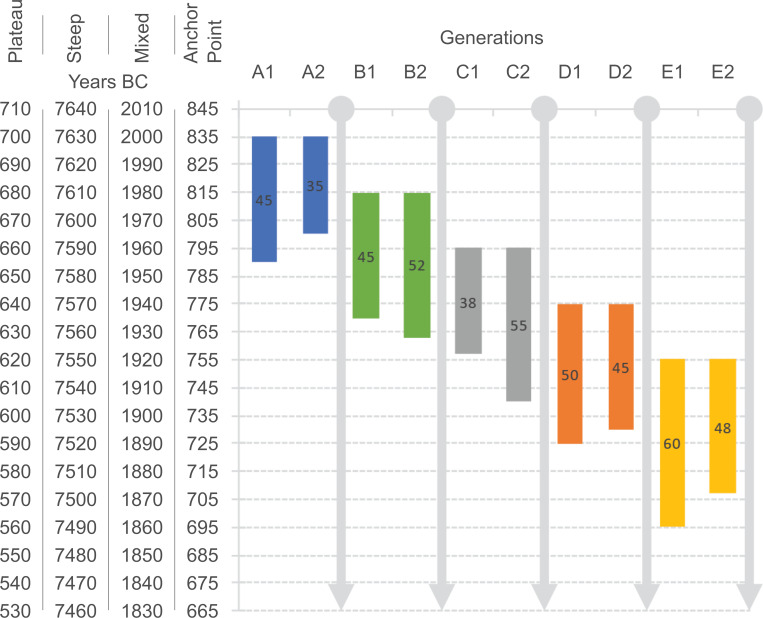
Graphical representation of the four simulated prehistoric single-strand pedigrees that are used for the simulations. A 20-year difference of YoB from one generation to another is prescribed. The AaD of each individual is indicated inside the coloured bars. The prescribed YoB for each of the general-type simulations (plateau, steep, mixed and anchor point) are shown on the left axis. The YoD is calculated from adding up YoB and AaD. The colour scheme of the generations is maintained in the following figures. The actual age parameters are listed in Fig 5.

All models are based on the same simulated five-generation single-strand pedigree (Fig 2) with only two adults per generation (35 to 60 years of age) (Fig 3). YoD of each pair of individuals per generation is earlier than the following one. Other, more complex pedigrees are not discussed here (S2 Fig), but their radiocarbon dates can be modelled in a similar way.

**Fig 4:**
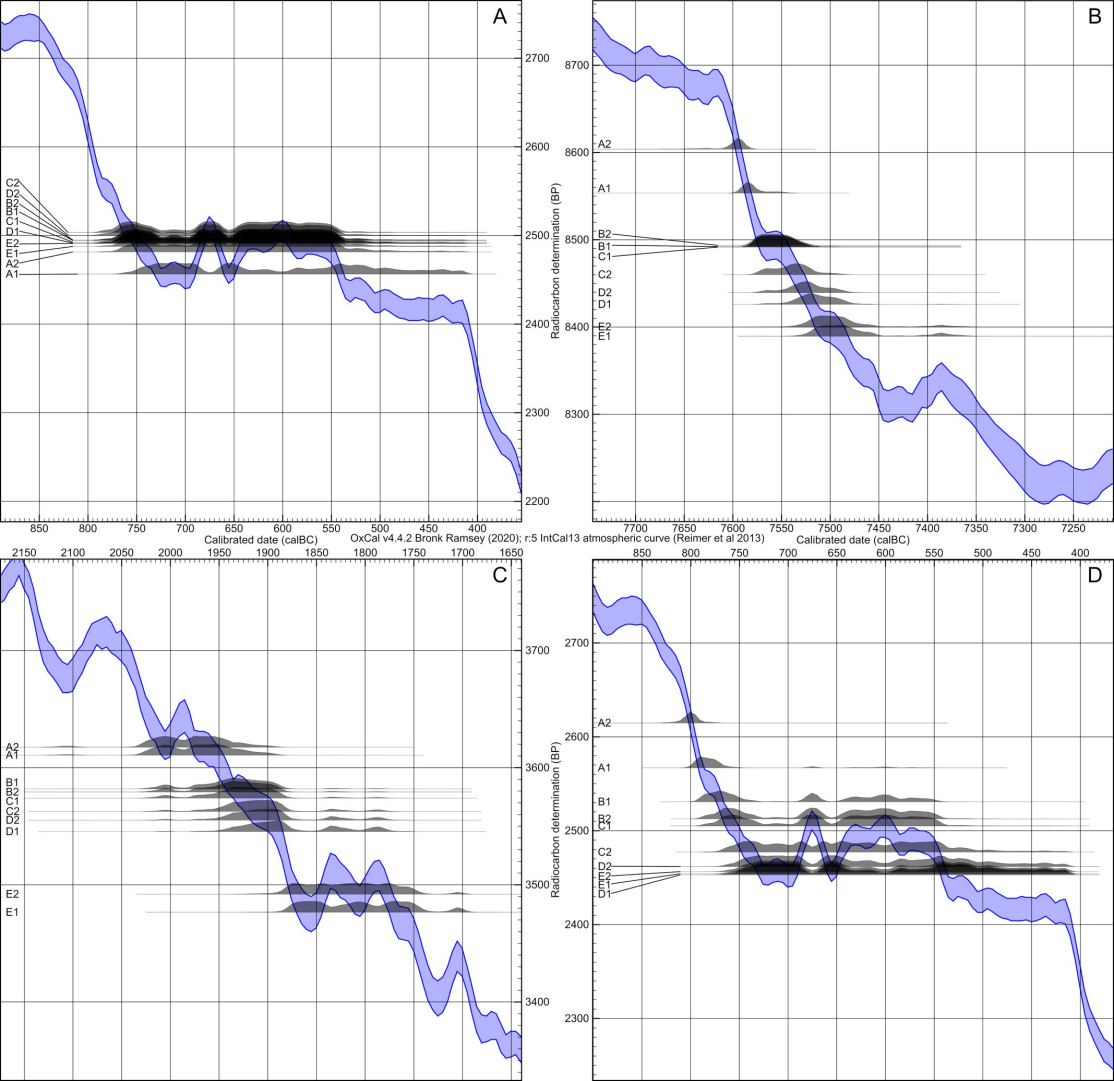
Calibration curves (IntCal13) and simulated dates of all four simulations. Plateau (A), steep (B), mixed (C) and anchor point (D) sections of the calibration curve (calibration results in Fig 5).

In addition, a fourth simulation (**Anchor Point**; Fig 1E and 1F) was created with potentially occurring radiocarbon dating patterns in pedigrees reconstructed from archaeological contexts to demonstrate the power of the Bayesian approach of combining multiple layers of evidence and information. This model consists of radiocarbon dates of the individuals from generations 2–5 falling in the Hallstatt plateau, whereas one of the dates from the first generation is within the steep section of the calibration curve right before the plateau phase (YoD individual A2: 800 BC) (Fig 4).

**Fig 5:**
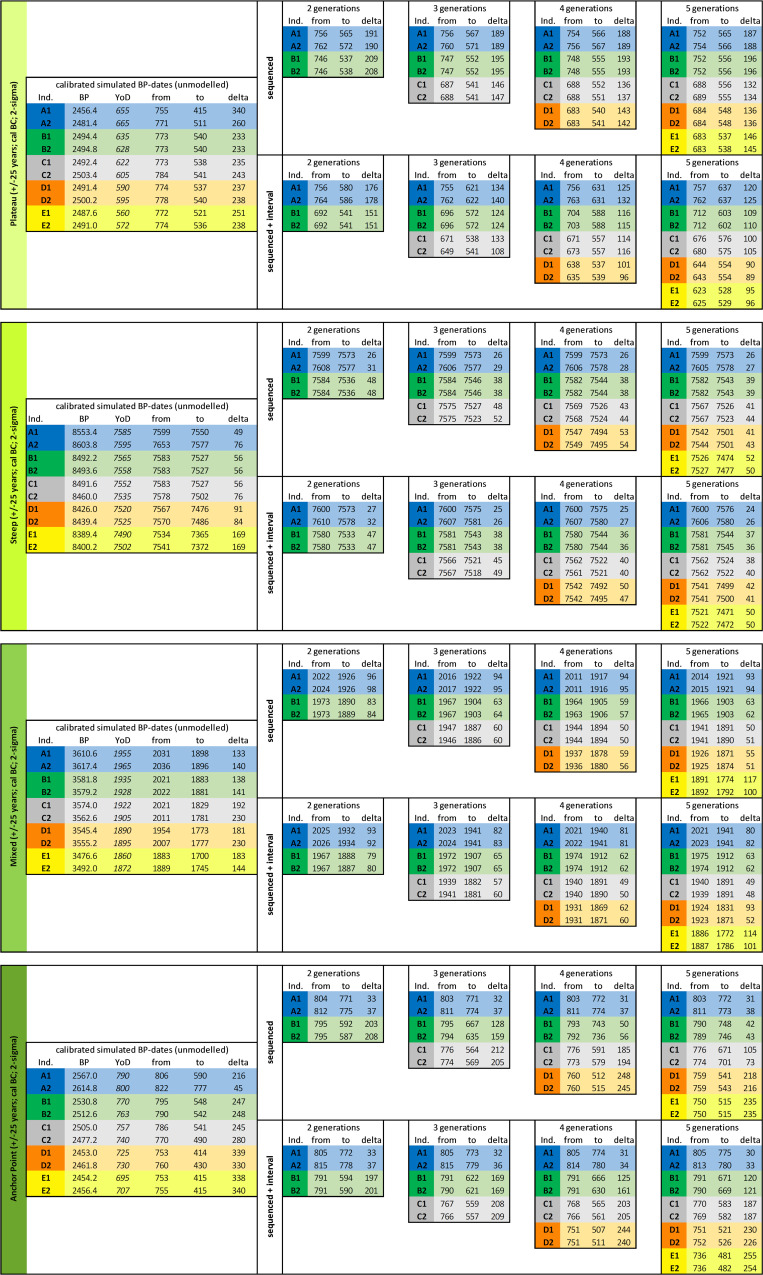
Results of Bayesian modelling of the radiocarbon dates for all four simulated pedigrees. Those include the unmodelled radiocarbon dates for each individual and their 2-sigma dating ranges as well as all results of runs with and without intervals between generations divided according to the varying number of generations.

**Fig 6:**
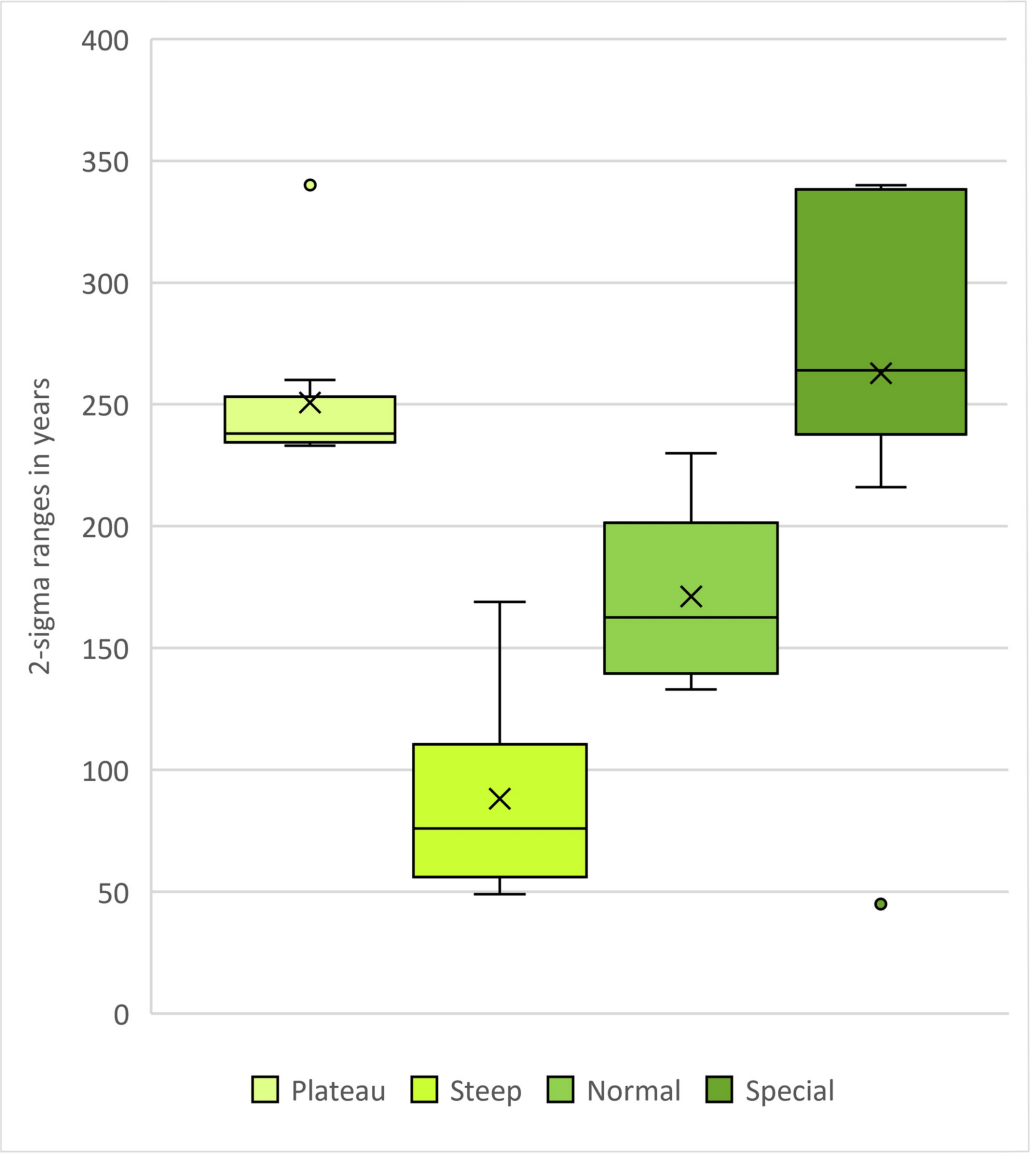
Whisker-Box-plots showing 2-sigma ranges of the calibrated dates used for the four pedigree simulations. Parameters and results in Fig 5.

Fig 4 shows the calibration time frames of the plateau-like (A: Hallstatt plateau), steep (B) and mixed section (C) of the calibration curve used for modelling, as well as the anchor point (D), with a pedigree spanning a steep and a plateau section. When calibrating the simulated ^14^C ages individually without any additional chronological information, dating ranges of the plateau-like section show intervals from around 230 up to 340 years (for the latter, see individual A1: 2456 ± 25 BP: 755–415 cal BC), whereas dates from the steep section are ideally constrained to only 49 years (e.g., individual A1: 8553 ± 25 BP: 7599–7550 cal BC), most of them below a 100-year margin. The latest calibrated dates of the steep calibration curve simulation already peripherally touch the next wiggle at about 7460 cal BC, extending their 2-sigma ranges up to 170 years. In the mixed section, date ranges fluctuate between 133 and 230 years, with an average of about 184. In the anchor point scenario, a single date shows a range of about 45 years because of its chronological placement before the plateau. All others have hugely increased ranges up to 340 years (c.f. Fig 6).

Sections of the calibration curve described as “mixed” do not show prolonged or pronounced plateaus or steep sections. They are characterized by rapid succession of wiggles and appear most frequently throughout the whole calibration curve (see Fig 1). The time frame for our modelling of a mixed section was set roughly between the ^14^C dates of 3600 and 3500 BP, with calibrated dates from 2030 to 1890 cal BC, reflecting the sections of the calibration occupied by the pedigrees of POST and OBKR in the case studies.

This particular plateau section was deliberately chosen for this model because of the vast amount of archaeological material and contexts situated at this particular period of time across Europe, in other words the (Early) Iron Age. Consequently, a successful application of Bayesian modelling of radiocarbon dates with pedigree-based priors will help refine chronological systems in the Early Iron Age. Often referred to as the Hallstatt plateau in general, it is noteworthy that the flat section of the calibration curve is divided into two sub-plateaus at around 540 cal BC, which is of significance for events whose durations are restricted to a time before or after 540 cal BC (cf. [36]). Equally important for the interpretation of radiocarbon dates within that time frame are the down-wiggles (troughs) during the first half of the “Hallstatt” plateau (Fig 4). In that case, a person’s YoD at 655 cal BC (individual A1 in this model) would have the same date range as a person who died in 710 or 540 cal BC.

Each of those simulations—which cover different shapes of the calibration curve—were run with different numbers of prescribed artificial generations (of known sequence), starting with 5 and going down to 2 generations. Therefore, the whole set of simulations includes effects of the calibration curve and the number of modelled generations. As an example, simulations were created using analytical uncertainties of 25 years of the ^14^C dates, which covers the typical uncertainty range of today’s ^14^C measurements (see [18]). For explanatory reasons, only results for 25 years of uncertainty are graphically displayed and discussed, because no substantial differences were observable between the varying uncertainties of 20, 25 and 30 years.

Running the simulations involved the following steps: (1) Constructing an artificial pedigree with predefined relationships and Year-of-Death (YoD) of each individual; (2) loocking up conventional ^14^C ages from the calibration curve that correspond to the YoDs; (3) building a Bayesian model that represents the predefined pedigree by sequencing the generations and using the conventional ^14^C ages of the individuals. OxCal’s “Sequence()” command is used to chronologically sequence the generations: thereby, the model will chronologically place generation 1 before generation 2 and so on, the time gap between them is not defined and can be 0 or more years. Individuals within the same generation are not treated in any chronological order. OxCal’s command “Phase()” is used in those cases. Additionally, the period of time between generations (Tgen) was prescribed using OxCal’s “Interval(N(Tgen,10))” command. The interval was individually calculated from the known average YoDs of the consecutive generations. An uncertainty of 10 years was prescribed to that interval in order to simulate a more realistic picture of the actual real-life situation, where the intervals between generations are not precisely known. The “interval” command is placed between phases rather than inside the “phase” commands in order to prescribe the time interval between generations. Sequence boundaries set the generations to be contiguous.

The OxCal code of the basic setup for the Bayesian models of the four simulations looks as follows (for a two-generation model) (c.f. S1 Codes):

Plot()

{

Sequence()

{

Boundary("Start Gen 1");

Phase("Gen 1")

{

R_Date("A1",A1,err);

R_Date("A2",A2,err);

};

[Interval(N(Tgen,10));]

Boundary("Gen 1/2");

Phase("Gen 2")

{

R_Date("B1",B1,err);

R_Date("B2",B2,err);

};

Boundary("End Gen 2");

};

};

### Case studies

#### Haunstetten—Postillionstraße (POST)

The excavated and documented part of the Early Bronze Age cemetery of Haunstetten contained at least 40 graves with 41 individuals [18]. Due to destruction in the 1990s, it was not possible to document the entire burial site. A total of 60 graves is estimated in its original condition. All individuals were inhumations and were buried along a N-S-axis in accordance with the gender-specific orientation and body positioning of the Bell Beaker and Early Bronze Age type. Type artefacts from the burials can be assigned to the first relative chronological sub-phase of the Early Bronze Age (Bz A1a), with no inner chronological order obtainable through archaeological assessment.

**Fig 7:**
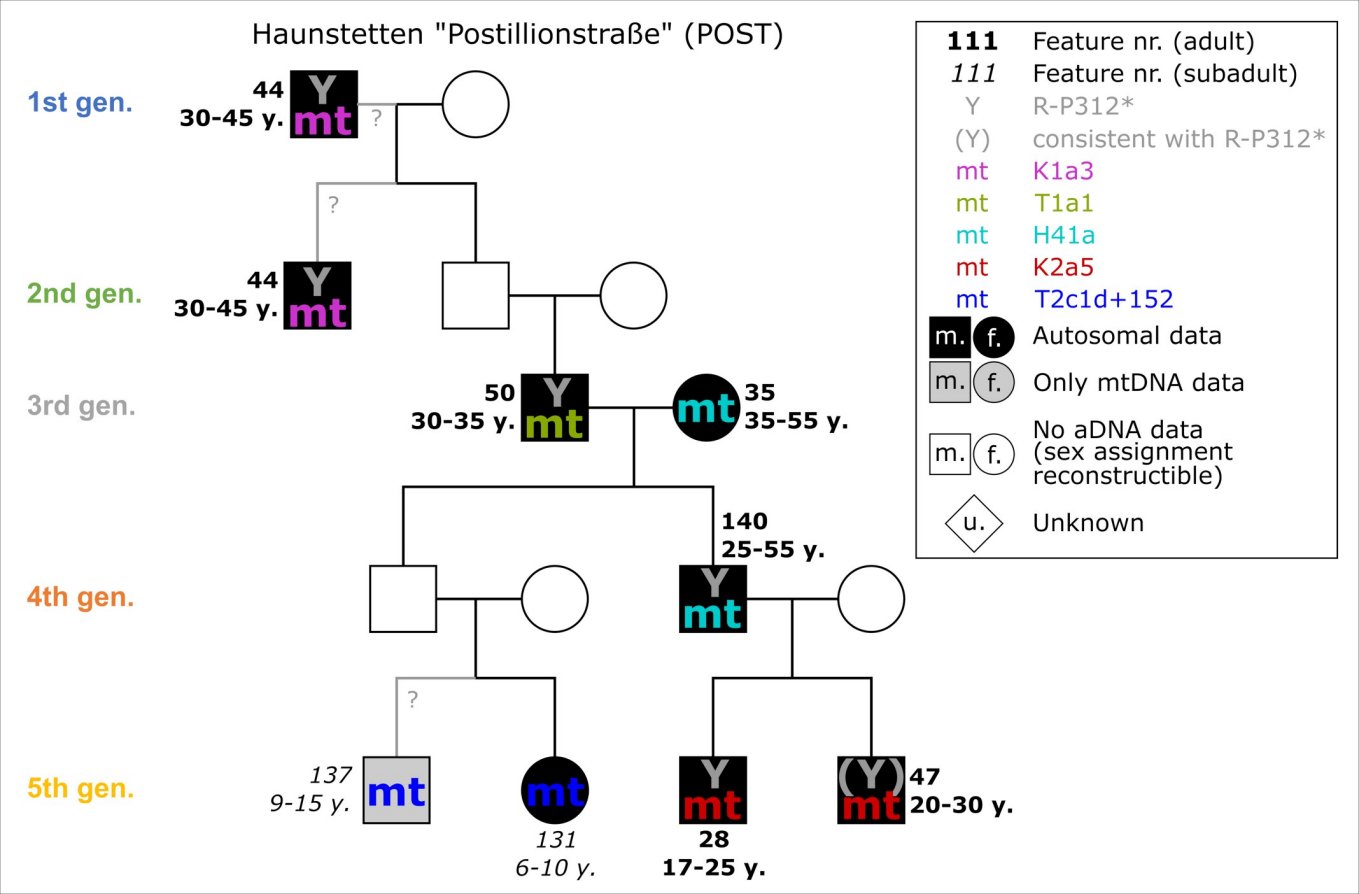
Pedigree of POST with four or five generations based upon evidence from aDNA analysis. Ancient DNA analysis includes the coefficient of relatedness, shared mtDNA haplotypes and Y-haplogroups. Feature number and AaD estimate are depicted alongside the symbol for each individual.

The skeletal remains of every individual were analysed anthropologically. Unfortunately, the gravel soil caused physical damage to bones, causing difficulties in determining the AaD (e.g., POST_140: 25–55 years). Most individuals were adults, with AaD ranging from 17 up to probably 55 years, and two individuals (POST_131, POST_137) at the end of the lineage died during childhood or youth (Fig 7).

22 individuals were sampled for DNA analyses, 19 of which provided mitochondrial genomes. 15 were selected for targeted nuclear DNA capture and relatedness analyses. A biological affiliation to the biological pedigree was detected for 10 individuals, and 7 of them could be placed in an explicit pedigree, with an additional individual added on the basis of the mitochondrial DNA. The pedigree consists of four or five generations, depending on the positioning of POST_44, who could be placed as either grandfather in the first or uncle in the second generation. After the third generation, the pedigree splits into two paternally related lineages. These individuals were buried in close proximity to each other in the northern half of the cemetery, partly occupying the prominent burial mounds, and were very well equipped with grave goods.

Radiocarbon dates without HBCO correction gathered from skeletal remains fall between 2197 and 1772 cal BC (2-sigma), with most of them between 2140 and 1880 cal BC, covering a small section of the calibration curve with wiggles (ca. 2140–2040 cal BC) and a steep section (ca. 2040–1880 cal BC). The radiocarbon ages of individuals from the pedigree lie between 3707 and 3608 BP with a standard deviation of 20–24 years and result in unmodelled calibrated dates from 2196 cal BC to 1906 cal BC (2-sigma), thus, 290 calendric years. The individual 2-sigma spans range from 121–188 years (Table 1).

**Table 1:** Basic information about the sampled individuals from the case studies POST, OBKR and AITI.

Site name	Site abbreviation	Feat. No.	Individual ID	Generation level	Labor No. (MAMS)	C-14 age	±	Unmodelled date cal BC (2-sigma)	Unmodelled timespan (2-sigma in calendric yrs.)	AaD-Classification	AaD in calendric yrs.	HBCO-corr.	BP-HBCO
Haunstetten–Postillionstraße	POST	44	POST_44	1^st^ or 2^nd^	18964	3681	23	2139–1979	160	Adultus (middle)—Maturus (young)	30–45	-15	3666
Haunstetten–Postillionstraße	POST	50	POST_50	3^rd^	18966	3707	24	2196–2029	167	Adultus (middle-old)	30–35	-15	3692
Haunstetten–Postillionstraße	POST	35	POST_35	3^rd^	18962	3621	20	2033–1918	115	Adultus (old)—Maturus (old)	35–55	-20	3601
Haunstetten–Postillionstraße	POST	140	POST_140	4^th^	18973	3631	20	2114–1926	188	Adultus (middle)—Maturus (old)	25–55	-10	3621
Haunstetten–Postillionstraße	POST	137	POST_137	5^th^	18971	3608	20	2029–1912	117	Infans II—Juvenis (young)	9–15	0	3608
Haunstetten–Postillionstraße	POST	131	POST_131	5^th^	18971	3635	20	2120–1938	182	Infans II	6–10	0	3635
Haunstetten–Postillionstraße	POST	28	POST_28	5^th^	18960	3608	20	2027–1906	121	Juvenis (old)—Adultus (young)	17–25	-5	3603
Haunstetten–Postillionstraße	POST	47	POST_47	5^th^	18965	3662	24	2134–1956	178	Adultus (young-middle)	20–30	-5	3657
									avg: 153.50				
Königsbrunn—Obere Kreuzstraße	OBKR	6	OBKR_6	1^st^	18890	3611	23	2029–1903	126	Juvenis	15–18	0	3611
Königsbrunn—Obere Kreuzstraße	OBKR	80	OBKR_80	2^nd^	18901	3664	24	2134–1959	175	Adultus (middle-old)	30–40	-15	3649
Königsbrunn—Obere Kreuzstraße	OBKR	86	OBKR_86	2^nd^	18907	3615	24	2032–1901	131	Adultus (middle-old)	30–40	-15	3600
Königsbrunn—Obere Kreuzstraße	OBKR	81	OBKR_81	4^th^	18902	3602	24	2024–1896	128	Infans I	4	0	3602
Königsbrunn—Obere Kreuzstraße	OBKR	82	OBKR_82	4^th^	18903	3600	24	2023–1894	129	Infans I	3	0	3600
									avg: 137.8				
Kleinaitingen—Gewerbegebiet Nord	AITI	87	AITI_87	1^st^	21588	3433	28	1876–1660	216	Adultus (middle-old)	30–40	-15	3418
Kleinaitingen—Gewerbegebiet Nord	AITI	86	AITI_86	2^nd^	21587	3480	34	1891–1694	197	Infans I	4–6	0	3480
Kleinaitingen—Gewerbegebiet Nord	AITI	119	AITI_119	2^nd^	21594	3470	27	1882–1696	186	Juvenis	13–17	0	3470
Kleinaitingen—Gewerbegebiet Nord	AITI	120	AITI_120	2^nd^	21595	3417	27	1866–1638	228	Adultus (middle)	30	-10	3407
									avg: 206.75				

#### Königsbrunn—Obere Kreuzstraße (OBKR)

The Early Bronze Age of Königsbrunn—Obere Kreuzstraße (OBKR), formerly called “Baugebiet 110” [37], was fully excavated and contained 48 graves with 50 individuals in total [18]. In contrast to Haunstetten, the cemetery of OBKR is divided into five groups of burials stretching along the N-S-axis; the grave pits have the same N-S orientation. All deceased were inhumated and buried in crouched positions. Just like in POST, all type artefacts can be assigned to the sub-phase Bz A1a without any further inner chronological order.

**Fig 8:**
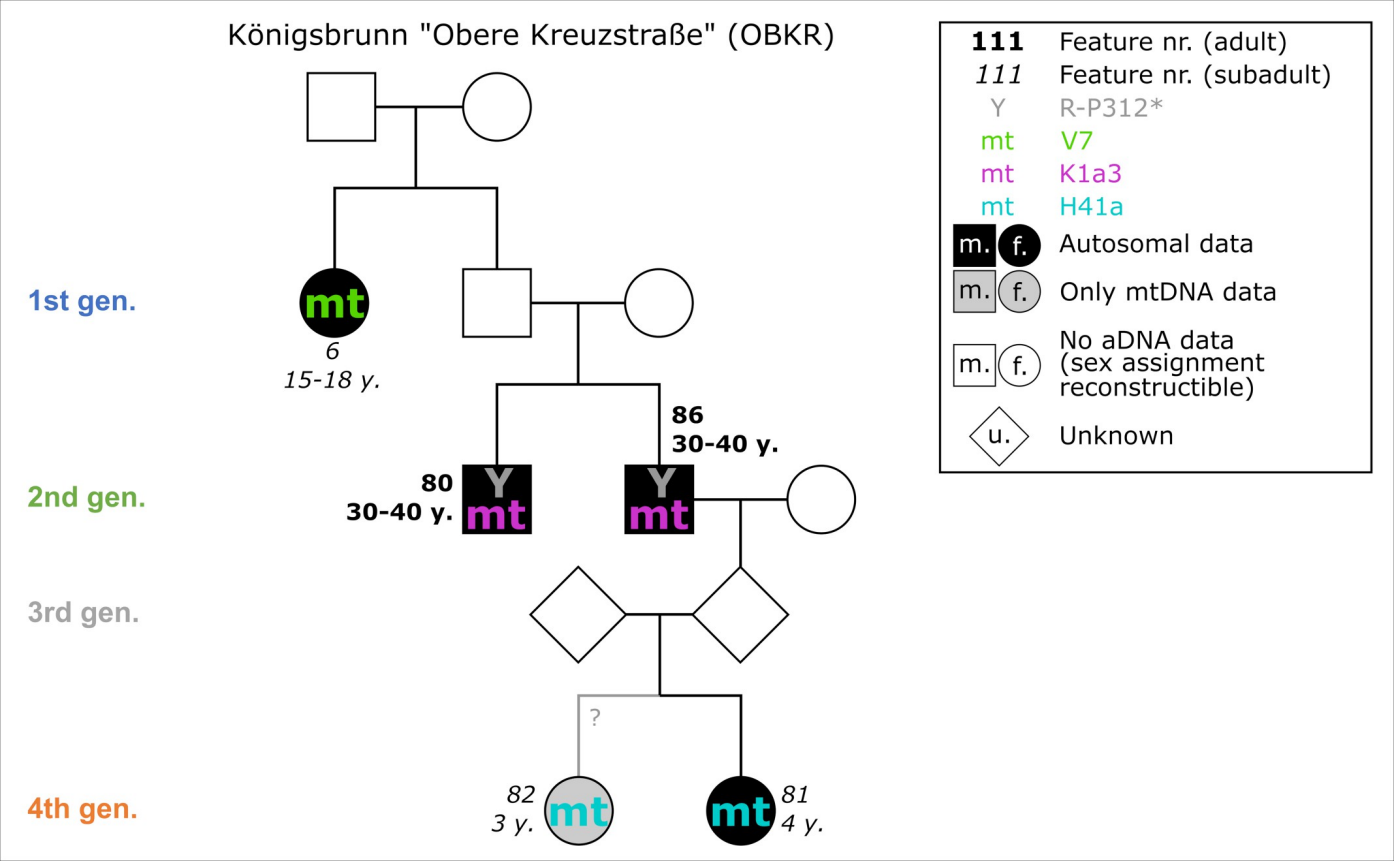
Pedigree of OBKR with four generations based upon evidence from aDNA analysis. Ancient DNA analysis includes the coefficient of relatedness, shared mtDNA haplotypes and Y-haplogroups. Feature number and AaD estimate are depicted alongside the symbol for each individual.

All skeletal material was anthropologically analysed [38]. Excellently executed modern excavation documentation and treatment of the skeletal remains balanced the physical damage caused by gravel soils. AaD determinations of individuals within the pedigree are constrained to 10 years at most. The two brothers of the second generation died as adults between 30 and 40 years, whereas OBKR_6 died as a juvenile or young adult and OBKR_81 and OBKR_82 did not live beyond early childhood (Fig 8).

All 23 of the sampled individuals yielded mitochondrial genomes. 18 were selected for nuclear capture. The pedigree spans four generations, with 4 individuals attributed to it through the analyses of nuclear DNA and an additional individual through mitochondrial DNA. One further individual is related to the biological family but cannot be explicitly placed in the pedigree. The subadult sisters 81 and 82 were buried side by side, whereas the adult brothers 80 and 86 were buried ca. 60 m apart from each other.

Skeletal remains of 24 individuals were radiocarbon dated. Calibrated, unmodelled dates without HBCO correction range between 2136 and 1780 cal BC (2-sigma). Most of them do not exceed the steep section of the calibration curve (until 1880 cal BC). The radiocarbon ages of individuals within the pedigree range between 3664 and 3600 BP with standard deviations of 23–24 years. They result in unmodelled calibrated dates from 2134–1894 cal BC with single 2-sigma ranges between 126 and 175 years (Table 1).

#### Kleinaitingen—Gewerbegebiet Nord (AITI)

The Early Bronze Age graveyard of Kleinaitingen—Gewerbegebiet Nord (AITI) contained 63 graves with a total of 72 individuals. The site was almost entirely excavated; only a part on the southern border is still preserved under a modern track. Apart from five cremations, two of them in biritual double burials, all graves contained inhumations in crouched positions aligned along the N-S-axis [18]. Three features contained triple burials: AITI_62, AITI_77 and AITI_115. The individuals in AITI_115 were cremated. Typo-chronologically speaking, the cemetery of AITI dates to the second sub-phase of Bz A1 (Bz A1b) until Bz A2(b).

**Fig 9:**
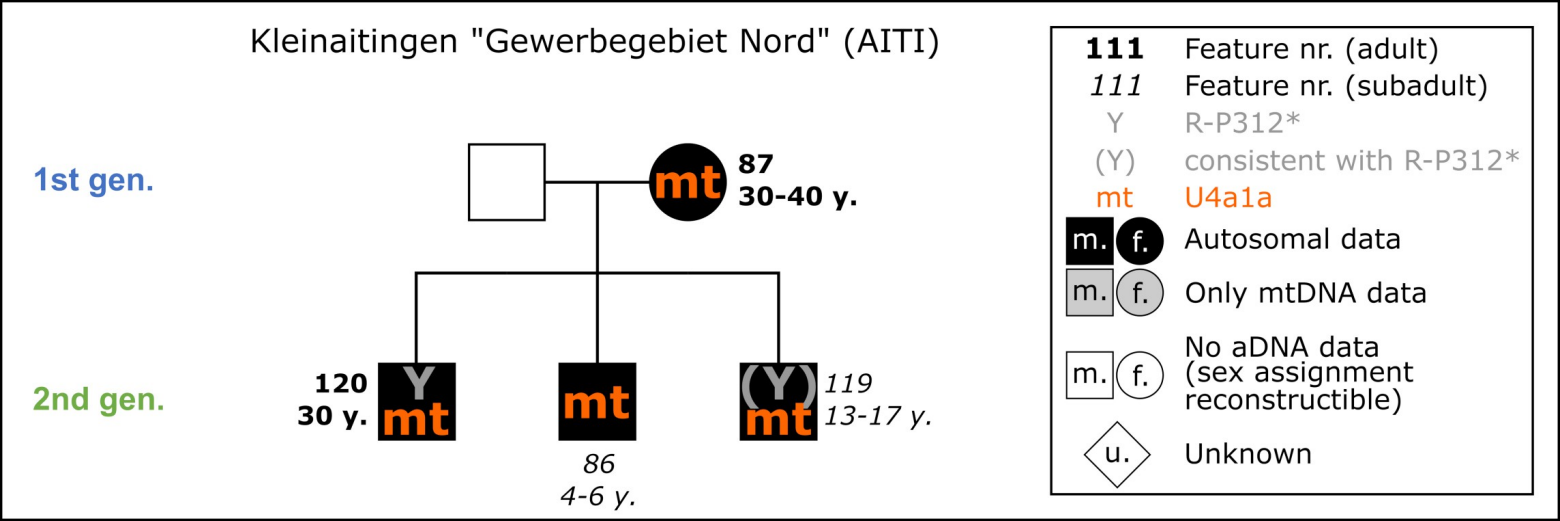
Pedigree of the nuclear family from AITI based upon evidence from aDNA analysis. The nuclear family includes a mother and her two subadult sons and an adolescent son. Ancient DNA analysis includes the coefficient of relatedness, shared mtDNA haplotypes and Y-haplogroups. Feature number and AaD estimate are depicted alongside the symbol for each individual.

The skeletal material was analysed anthropologically in its entirety [38]. Due to the calcareous gravel into which the graves were dug, frequently up to a large depth, the skeletal remains were chemically and physically well preserved. AaD uncertainties do not exceed 10 years. Two individuals of the second generation died as subadults, whereas AITI_120 reached 30 years of age (Fig 9).

**Fig 10:**
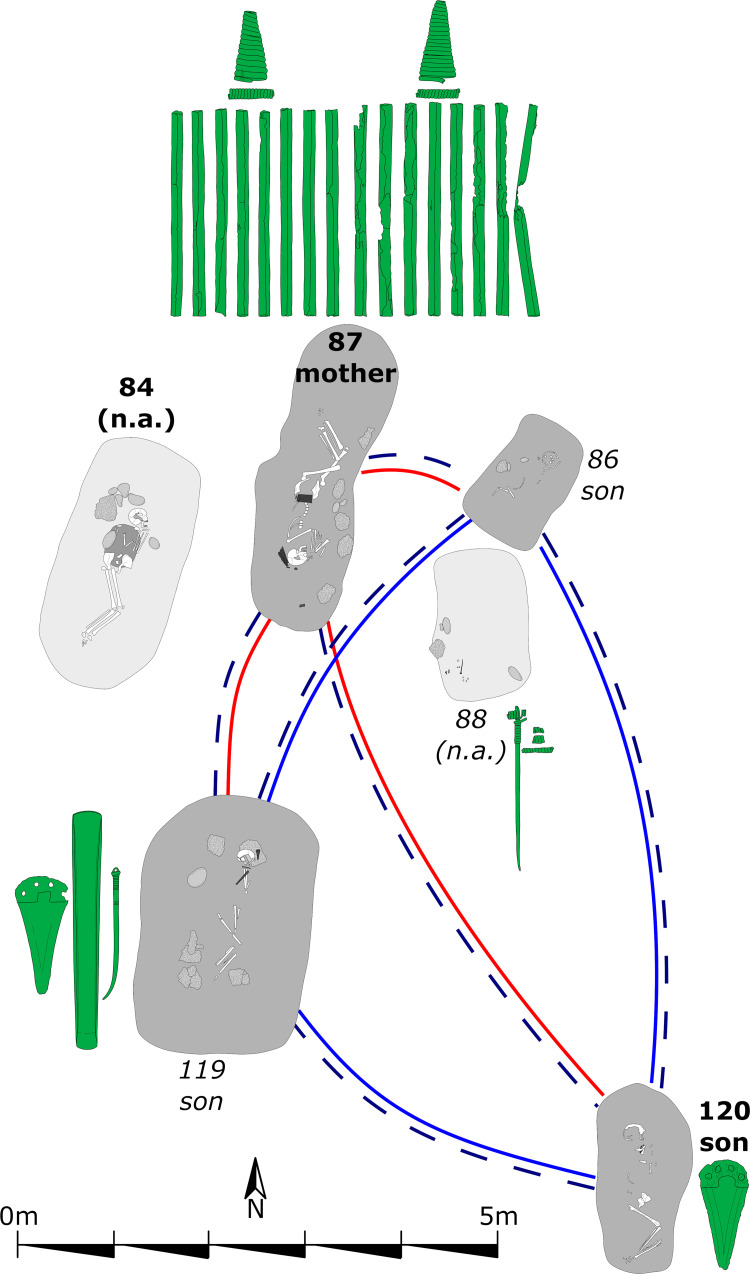
Detailed plan of the nuclear family of AITI with associated grave goods. Bold labels: Adults; italic labels: Subadults. Light grey shaded grave pits: Not analyzed (n.a.). Solid lines between individuals: First degree relationship—parent-offspring (red) and siblings (blue). Dashed line between individuals: mtDNA haplotype-sharing (U4a1a) indicating maternal lineage.

31 individuals were sampled for targeted DNA capture, and 27 provided sufficient data for kinship analysis. We find one mother (AITI_87) with three sons (AITI_120, AITI_86 and AITI_119) (Fig 9). Additionally, two other pairs of first-degree kinship were detected: father and son of unknown order (AITI_70 and AITI 72) and two brothers (AITI_43 and AITI 55). These families are more distantly related to each other, as well as to other individuals from the site, but cannot be placed in an explicit pedigree. All closely related families were buried next to each other, displaying their biological kinships (Fig 10). Because of its proximity, the unanalysed burial of a male adult (AITI_84) could be the father of the three sons, the biological partner of AITI_87 respectively.

Radiocarbon dates were obtained from skeletal remains of 35 individuals. The calibrated, unmodelled dates without HBCO correction range between 2116 and 1505 cal BC (2-sigma). Most of them cover the period between 2040 and 1700 cal BC. Five dates show ranges as late as ca. 1500 cal BC. The four individuals of the small biological pedigree delivered radiocarbon ages ranging from 3480 to 3417 BP with standard deviations of 27–34 years. Their calibrated dating ranges stretch from 1891–1638 cal BC (2-sigma), mainly covering two wiggle-sections of the calibration curve (ca. 1880–1770 cal BC and 1770–1670 cal BC). The individual ranges span between 186 and 228 years (Table 1).

## Results and discussion

### Simulations

#### Plateau model

**Fig 11:**
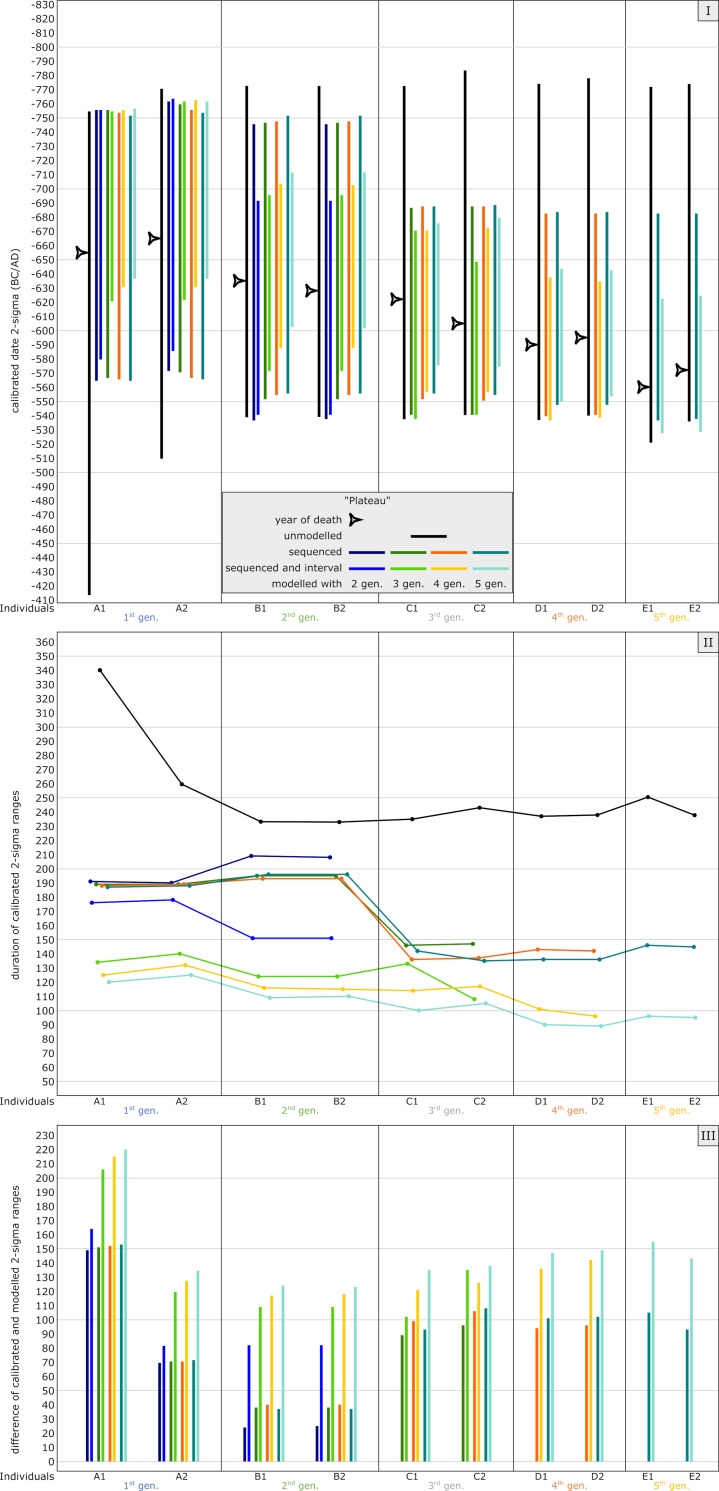
Graphic representation of the results of the simulated plateau model. Unmodelled, calibrated dates (black bars) plotted against modelled ranges of simulation runs with 2 (dark blue), 3 (green), 4 (orange) and 5 (light blue) generations incorporated into the models. The darker colour shade presents a pure sequencing model of each run, with the lighter colour shade indicating sequenced models and with defined intervals between the generations. Triangle symbols depict the actual YoD’s as inserted at the outset of the modelling process. (I) Dating ranges and YoD’s according to their calendric years. (II) Duration of calibrated unmodelled and modelled dates. (III) Calibrated range differences between unmodelled and modelled dates. For results and parameters of the simulation, cf. Fig 5, S1 Table and S1 Codes.

Fig 11 Panel I shows the results of the unmodelled simulated ^14^C ages (black bars), modelled without interval between generations (darker colored bars) and modelled using known intervals (lighter colored bars) compared to the prescribed YoD (black arrowhead) of the individuals. Models were rerun with five down to only two generations within a pedigree as priors. Already, the unmodelled calibrated date ranges of most individuals of the pedigree are restricted to a time before 510 cal BC. Only the dating range of A1 stretches beyond, even spanning the entire Hallstatt plateau. The unmodelled dating range of A1 stretches as far as 415 cal BC (Fig 5 and S1 Table).

All executed models with varying priors and generation levels cut off date ranges at 540 cal BC. Running the modelling process with five generations, all ranges end even before 550 cal BC except for individuals of the last generation, due to bracketing effects. The bracketing effect is an extended constraint of a modelled radiocarbon date, when there is at least one phase before and one after in the Bayesian model. After sequencing only the first two generations, the overall outcome of dating ranges seems to indicate the given/true order of YoD of the individuals, with modelled dating ranges of A1 and A2 stretching from around 760 cal BC to 565 cal BC: a major improvement, especially for individual A1. All simulations exclude a possible dating of the individuals C1-E2 before the down-wiggle of the calibration curve at around 700 cal BC, as observed in the unmodelled dates, and also eliminate the possibility of A1 dating to the second half of the Hallstatt plateau. All known YoD’s are located within the modelled dating ranges, indicating a successful modelling process, which is also true for the other three model simulations (steep, mixed and anchor point).

Adding a generation interval to the models constrains the calibrated ages even further. This is due to the additional information of chronological minimal distance between the generations, cutting off the dating range on the older or younger side. Especially the first two and the last generations benefit from this additional information/prior.

As mentioned before, all modelled dates get significantly constrained, especially that of A1. Its original range of around 340 shrinks to 187 years after sequencing, or even down to 120 years with additional intervals, an improvement of 153 years (45%) or 220 years (65%) (Fig 11 Panel II). The dating ranges of generations 2–4 were shortened by 24–105 years without intervals or 82–156 years with intervals. Many of the eight model runs (four generation level variations, each with and without intervals) resulted in upward bound ends of the lines, representing modelled date ranges. Those are caused by the lack of the bracketing effect, where no subsequent stratigraphic information is available, thus representing the latest generation level of each pedigree. Generally speaking, a pedigree with five generations comprises three generations (2^nd^-4^th^) on which bracketing has effect. If an individual or a generation is already affected by the bracketing effect, adding more generation levels did slightly improve the modelled ranges. In this example, the improvements of B1 and B2 stayed almost stable after a three-generation simulation (Fig 11 Panel II, green line), as well as C1 and C2 after a four-generation model (Fig 11 Panel II, orange line). In our setup, the modelled date ranges of individual A1 improved the most, with up to 220 years, while those of A2 were constrained ca. half as much (Fig 11 Panel III).

**Fig 12:**
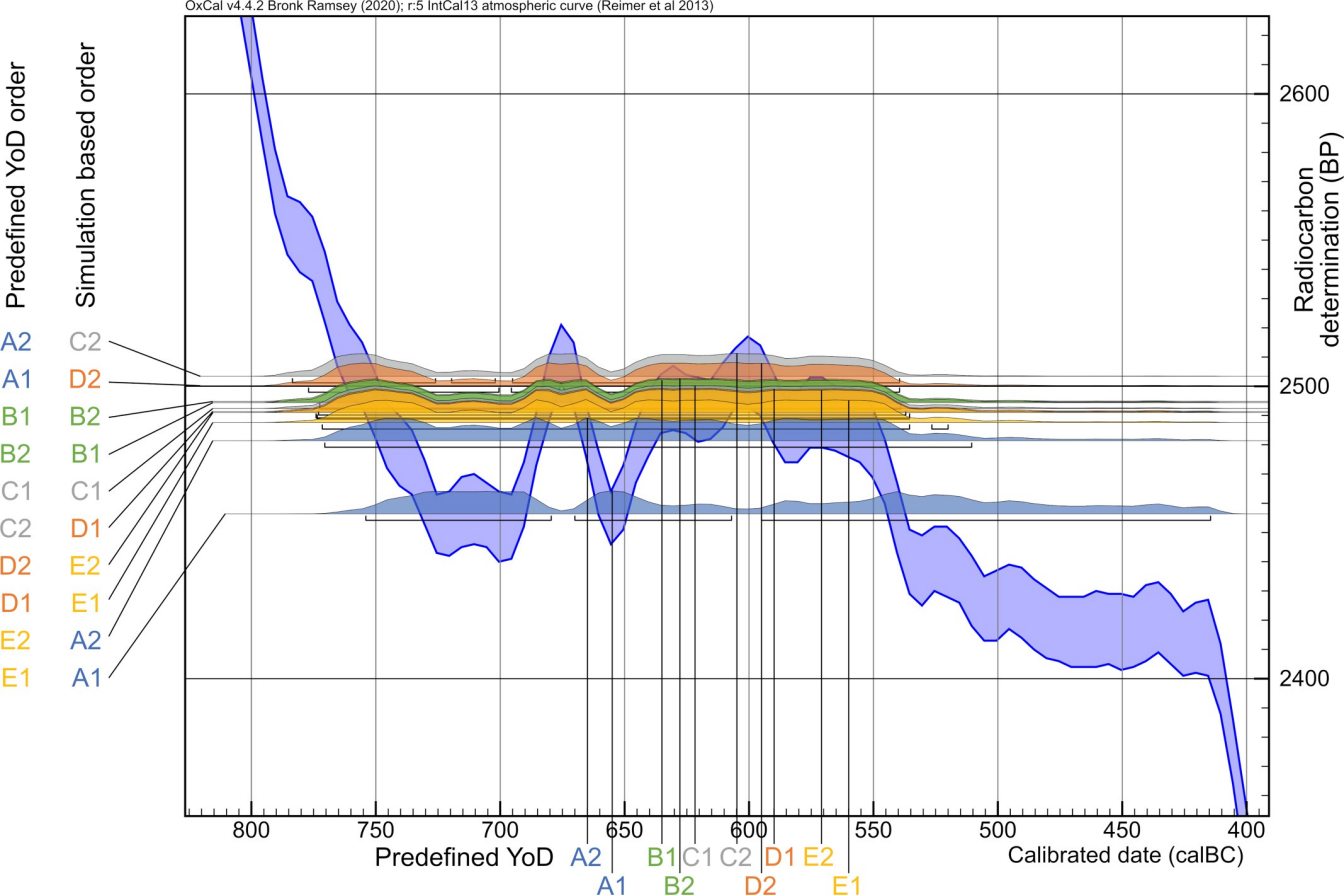
Closeup of calibrated radiocarbon dates of the plateau model. Unmodelled ^14^C ages with 25 years uncertainty on the Hallstatt plateau section (cf. Fig 4A) of the calibration curve (IntCal13) show the inversion of calibrated dates (2-sigma), especially individual A1 (YoD = 655 BC), resulting in a much wider dating range and in the youngest placement regarding simulation-based orders.

Inversions or permutations, a seeming reversal of the order of dated events, are due to down-wiggles in the calibration curve, as seen with individual A1 (Fig 12). The YoD of A1 coincides with a trough/valley at 655 BC. The measured, or in this model case, predefined, radiocarbon age, with its uncertainty range of ± 25 years, also touches the wiggle before and both plateau sections after 655 BC, resulting in a wide calibrated date range. Except for A2, all other YoDs are younger. Their YoDs coincide with the upper peaks and plateau section and therefore only match the first half of the Hallstatt plateau.

The plateau model exemplifies the potential and limits of Bayesian modelling of a series of events (subsequent deaths of individuals within a pedigree) when the overall period of interest is shorter than the plateau itself and no other chronological information is available (cf. [5]). Without the results of aDNA analyses or other chronologically relevant information, the inner chronological order would not have been recognisable, and individual A1 could potentially have been the youngest. However, the reduction of calibrated 2-sigma ranges of up to 220 years shows the advantages of the particular Bayesian approach applied in this study, which can be essential for archaeo-chronological interpretations. In addition, it eliminates inversions of unmodelled datings, even modelling with just two generations (see below). In this model setup, for example, the dating ranges were drastically reduced, almost entirely excluding a chronological assignment of any individual to the younger subphase of the Hallstatt plateau beginning at around 540 cal BC.

#### Steep model

The time frame for the steep model (around 7500 cal BC) was chosen because of its long, steep section in the calibration curve, which causes very narrow calibrated date ranges even for unmodelled ^14^C ages. Most calibrated dates of the individuals are placed within the steep section, while the latest generation (E1 and E2) show unmodelled calibrated dates that are affected by the wiggle between 7470 and 7330 cal BC (see Fig 4B). In contrast to the anchor point model, where one individual anchors the time frame of the entire pedigree, generations 1–4 are here basically the fixpoint for the last generation, with a low precision of calibrated, unmodelled radiocarbon dates (Fig 5 and S2 Table).

The dating range of A1 is limited to 49 years (8553.4 ± 25 BP: 7585–799 cal BC), whereas those of E1 and E2 stretch over 169 years (e.g., E1: 8389.4 ± 25 BP: 7534–7365 cal BC). Unmodelled and calibrated range probabilities of A2-D2 vary from 49 to 91 years.

**Fig 13:**
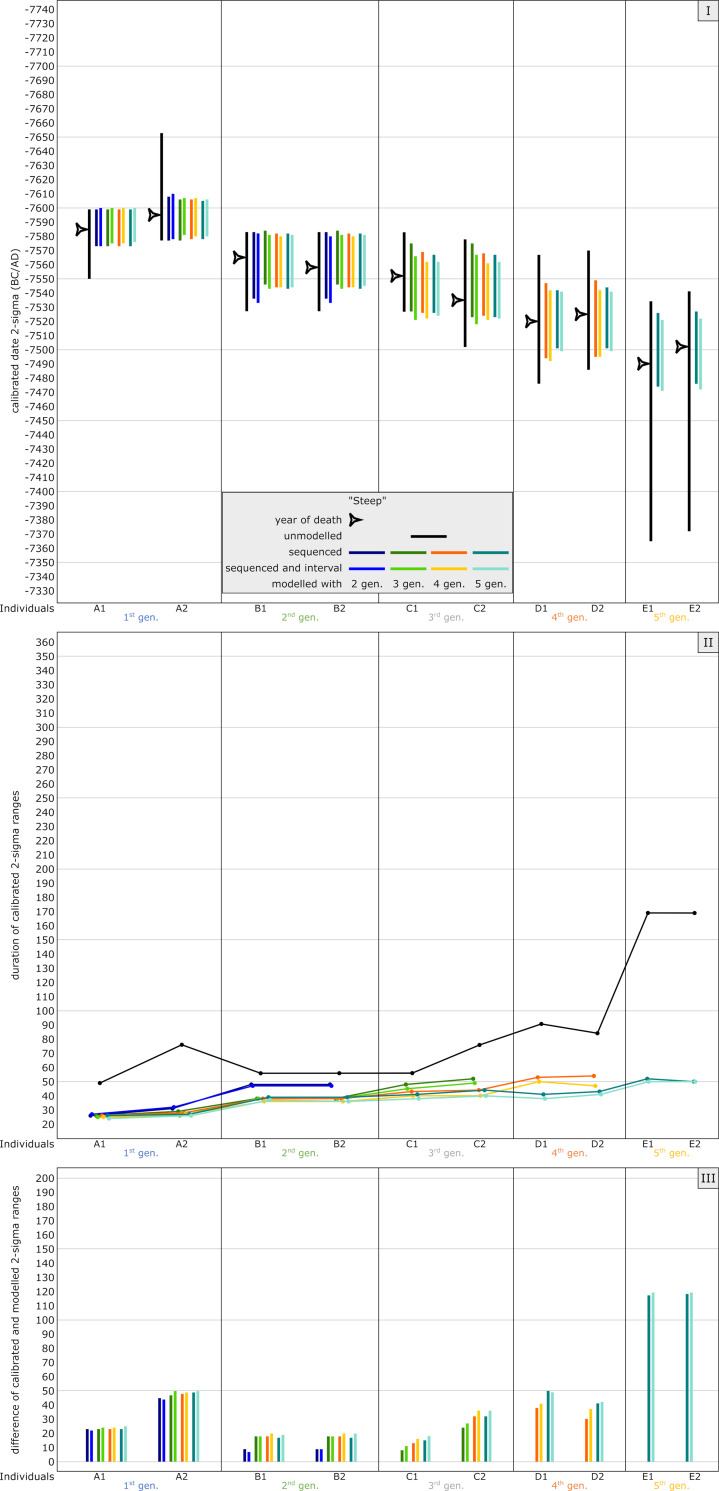
Graphic representation of the results of the simulated steep model. Unmodelled, calibrated dates (black bars) plotted against modelled ranges of simulation runs with 2 (dark blue), 3 (green), 4 (orange) and 5 (light blue) generations incorporated into the models. The darker colour shade presents a pure sequencing model of each run, with the lighter colour shade indicating sequenced models and with defined intervals between the generations. Triangle symbols depict the actual YoD’s as inserted at the outset of the modelling process. (I) Dating ranges and YoD’s according to their calendric years. (II) Duration of calibrated unmodelled and modelled dates. (III) Calibrated range differences between unmodelled and modelled dates. For results and parameters of the simulation, cf. Fig 5, S2 Table and S2 Codes.

Our Bayesian approach already shows significant improvements when modelling with only the first two generations (Fig 13 Panel I). Because of the narrow dating ranges even without modelling, adding more generations after the second does not lead to substantial improvements. This is clearly visible for individuals A1-C2. In this setup, no significant differences can be observed concerning a simple sequence-model and the model that includes generation intervals. The shape of the calibration curve can already place sufficient constraints on the calibrated dates.

Taking the five-generation model as a baseline, modelled dating ranges lie between 24 and 52 years, which is an incredible accuracy for absolute dating methods used in archaeology, apart from dendro-chronology (Fig 13 Panel II). Range probabilities of A1 and A2 with 24 and, respectively, 26 years do not even substantially exceed the time span of a generation gap (Δt YoB). The bracketing effect is obvious with each last generation of the particular model but with values of 12 years or less. After the bracketing effect is active, no further improvement of modelled dates is observable, similar to the plateau simulation.

Improvements differ quite substantially between the generations because of the aforementioned wiggle after 7470 cal BC, which affects only individuals E1 and E2 (Fig 13 Panel III). The overall improvement average, based upon the five-generation setup (solely sequenced), is 51,5 years. The overall average for individuals entirely within the steep section lies at 33,75 years (28,57–69,74%), whereas date ranges of E1 and E2 are shortened by 119 years, which is an enhancement of 70%.

#### Mixed model

**Fig 14:**
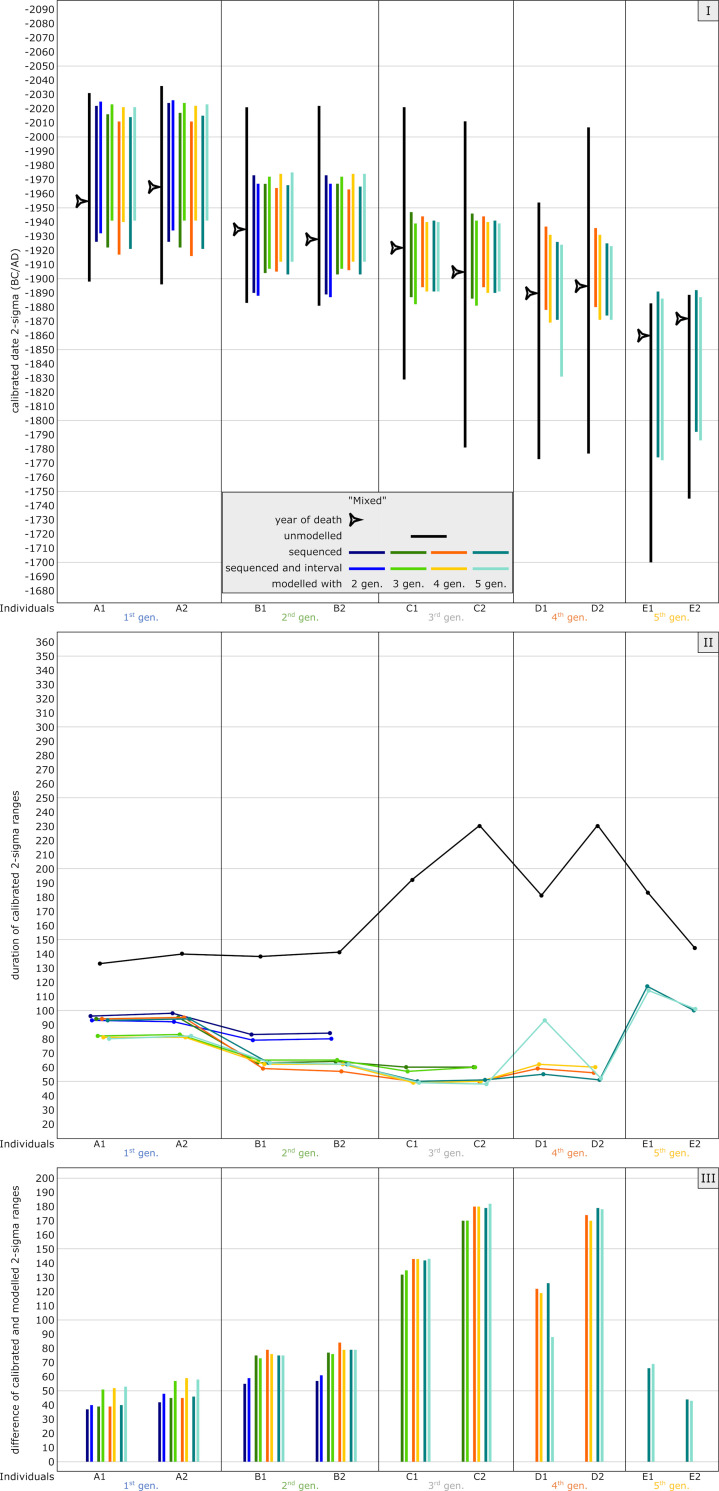
Graphic representation of the results of the simulated mixed model. Unmodelled, calibrated dates (black bars) plotted against modelled ranges of simulation runs with 2 (dark blue), 3 (green), 4 (orange) and 5 (light blue) generations incorporated into the models. The darker colour shade presents a pure sequencing model of each run, with the lighter colour shade indicating sequenced models and with defined intervals between the generations. Triangle symbols depict the actual YoD’s as inserted at the outset of the modelling process. (I) Dating ranges and YoD’s according to their calendric years. (II) Duration of calibrated unmodelled and modelled dates. (III) Calibrated range differences between unmodelled and modelled dates. For results and parameters of the simulation, cf. Fig 5, S3 Table and S3 Codes.

The simulated YoD’s of the individuals in this model setup (around 1900 cal BC) were chosen to be similar to the real case studies of the Early Bronze Age pedigrees discussed in this paper, for which some information is missing (e.g., missing generation levels) or which have a higher uncertainty (AaD-estimation). The unmodelled, calibrated dates fall onto two small wiggles (2040–1940 cal BC and 1880-1770/1750 cal BC) and a short steep section of the calibration curve in between them (see Fig 4C). Date ranges of individuals A1-B2 end before ca. 1880 cal BC, with C1-D2 mainly overlapping both the steep section and upcoming wiggle, whereas E1 and E2 are fully situated within the second wiggle, with date ranges beginning at 1880 cal BC and ending around 1700 cal BC (Fig 14 Panel I). None of the dates really serve as an anchor point for the pedigree. The widest 2-sigma ranges can be observed for individuals C2 and D2. Unmodelled and calibrated range probabilities vary from 133 to 230 years (Fig 5). Datings of the entire pedigree stretch from 2036–1700 cal BC, which is not sufficient for archaeological fine chronology without using additional information (Fig 5 and S3 Table).

Adding information from aDNA results in substantial constraints for almost every individual in this simulation. Even running the modelling process with solely the two first generations, a significant improvement is visible, especially for the dating ranges of the second generation, which now do not begin before ca. 1970 cal BC (B1 and B2). The bracketing effect is visible for all intermediate generations (gen. 2–4). Adding more generations down the pedigree does not show any enhancements thereafter. All individuals of the 3^rd^, 4^th^ and 5^th^ generations show significant constraints when modelled according to their pedigree-placement.

Within this model setup, no substantial differences can be observed between sequenced and additional generation-gap modelling (Fig 14 Panel II). The modelled radiocarbon dates of the first generation become constrained a bit more with each generation level added to the pedigree. In general, modelling the entire five-generation pedigree leads to maximum dating ranges of 117 years, with generations in between the first and last generation not exceeding 63 years, except from individual D1, when adding an interval in the five-generation setup. Radiocarbon dates of C2 are even narrowed down to 48 years at most. This is of major importance for archaeological dating. Similar to the plateau simulation, the bracketing effect is clearly visible for all intermediate generations. Adding even a sixth generation level, either with one, two or more individuals, would have the same bracketing effect on individuals E1 and E2.

Due to the wiggles and small plateaus in the calibration curve, the radiocarbon dates of C1-D2 resulted in wide dating ranges. Because of this, constraints are the highest for those four individuals (Fig 14 Panel III). While the dates of A1, A2 and E2 could be improved by approximately 50–60 years, the dating ranges of C1-D2 could be narrowed down substantially by up to 183 years.

#### Anchor point model

**Fig 15:**
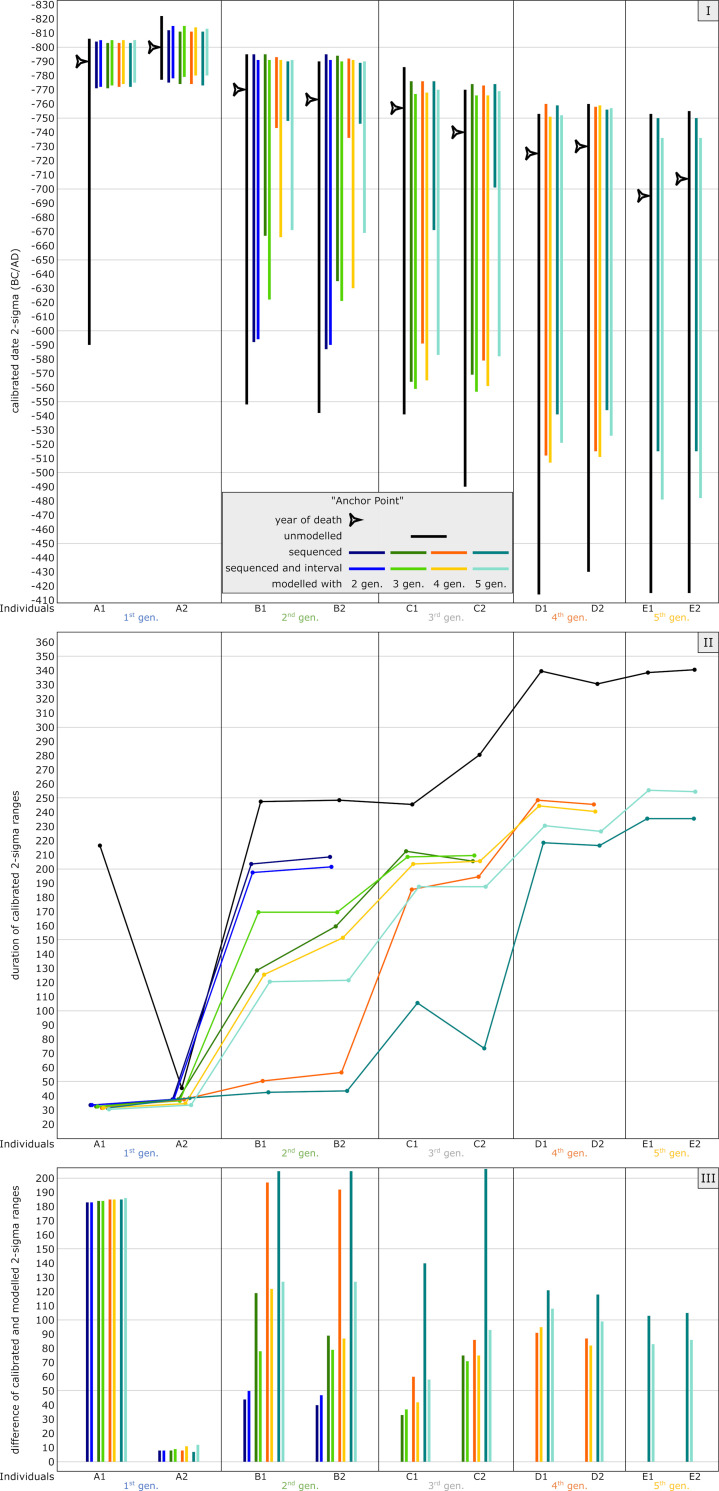
Graphic representation of the results of the simulated anchor point model. Unmodelled, calibrated dates (black bars) plotted against modelled ranges of simulation runs with 2 (dark blue), 3 (green), 4 (orange) and 5 (light blue) generations incorporated into the models. The darker colour shade presents a pure sequencing model of each run, with the lighter colour shade indicating sequenced models and with defined intervals between the generations. Triangle symbols depict the actual YoD’s as inserted at the outset of the modelling process. (I) Dating ranges and YoD’s according to their calendric years. (II) Duration of calibrated unmodelled and modelled dates. (III) Calibrated range differences between unmodelled and modelled dates. For results and parameters of the simulation, cf. Fig 5, S4 Table and S4 Codes.

This model presents a combined case of the plateau and the steep model, anchoring the pedigree with individual A2 (which died the earliest) to a steep section (Fig 1E), while all other individuals died during the subsequent plateau phase (Fig 1F). The differences in dating ranges are clearly visible with the unmodelled dates. The 2-sigma range of individual A2 is restricted to only 45 years, whereas the others show values of 216–340 years (Fig 15 Panel I).

Running the Bayesian model with two generations (with and without intervals) already reduces the probability span of A1 significantly from 216 down to 33 years, an improvement of 85%. With regard to the second generation, only a reduction of around 18% can be observed. The five-generation simulation produces substantial shortenings for at least the first two generations (e.g., B1, with 205 years reduction without integrating intervals in the model, which is an improvement of ca. 83%), whereas the reduction of dates of the following generations is more restricted. At least an unambiguous assignment to the first half of the Hallstatt plateau, or even before, can be made for the individuals down to D2 (Fig 5 and S4 Table).

The structure of the vast plateau section with its ups and downs is also responsible for the substantial differences seen between a model setup solely with a sequence and a sequence in combination with a generation interval (compare individuals B1 and B2 in the plateau model). Here, these differences can be clearly observed for all individuals that are affected by the bracketing effect. This is most obvious for individuals B1-C2. All individuals in the last generations are thus not influenced by any bracketing and show similar values with or without intervals (Fig 15 Panel II).

A bracketing effect is quite visible for all individuals framed by other generations. However, in contrast to the other three simulations, the bracketing effect enlarges with the number of subsequent generations, meaning the constraints of modelled dates increase with every simulation run with a generation level added. Here, the effect is best visible at the second generation (individuals B1 and B2). For example, the dating ranges of individual B1 show subsequent improvements of 17.8%, 36.9%, 60.9% and 16%, with a total reduction of 83%, in the simulation without intervals and 20.2%, 14.2%, 26% and 4%, with a total reduction of 51.4%, in the simulation with intervals.

This observation is caused by the course of the calibration curve, with its peaks and lows over the entire length of the plateau, and the particular chronological placement of the pedigree members (Figs 15 Panel III and 16). With every subsequent generation level added to the Bayesian model, the tail ends of the dating ranges get shortened: for example, with a four-generation model, the peak in the calibration curve at ca. 670 cal BC is still relevant for the outcome of the modelled date of individual B1. Adding yet another generation level erases this dating possibility (without intervals), resulting in a modelled radiocarbon date that ends at around 750 cal BC, which is within the steep section of the calibration curve (Fig 5). When adding intervals, the modelled dates can be shifted/forced into areas of the calibration curve with plateaus. Hence, calibrated dates can span wider periods of time. This is purely caused by the shape of the calibration curve in that particular period of time.

**Fig 16:**
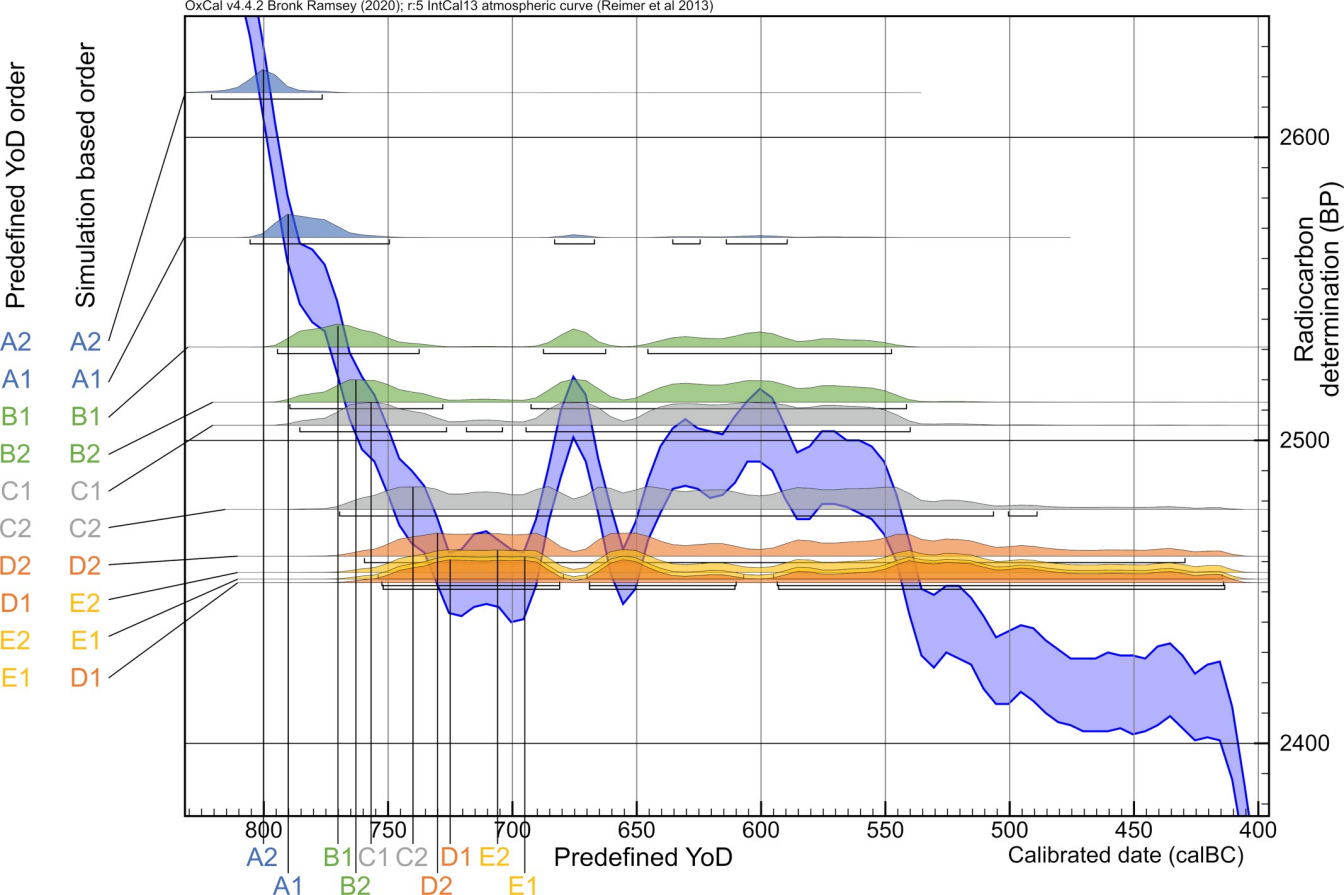
Closeup of calibrated radiocarbon dates of the anchor point model. Unmodelled ^14^C ages with 25 years uncertainty right before (individual A2) and on the Hallstatt plateau section (compare Fig 4A) of the calibration curve (IntCal13) show the inversion of calibrated dates (2-sigma).

The same inversion as seen in the plateau model with individual A1 can also be observed for individuals, whose real YoD would fall in the first down-wiggle (trough) between 745 and 690 cal BC. Here, this is demonstrated by individuals C2-E2 in the anchor point simulation. The calibrated dates of individuals A1 and B1-C1 only scratch the peaks and upper parts of the first half of the Hallstatt plateau, whereas C2-E2 coincide with the down-wiggles and both plateau areas, resulting in dating ranges of up to 340 years (2-sigma). In general, more individuals are aligned in the correct order of their YoD, due to the steep section of the curve at the beginning. Counterintuitively, though, with the pedigree in this anchor point scenario shifted 135 years backwards in time compared to the plateau simulation, more individuals (4 out of 10) seem to date even later, with date probability ranges up to 414 cal BC. This graphic should also make one aware of the positioning of the actual YoD of a sample compared to the unmodelled dating probability ranges, as unmodelled radiocarbon dates are still one of the main chronological estimates used in archaeology. Except for individual A2 in this case, the true YoD lies at least within the first fifth of the dating range, and for most of them even within the first tenth (e.g., D1).

**Fig 17:**
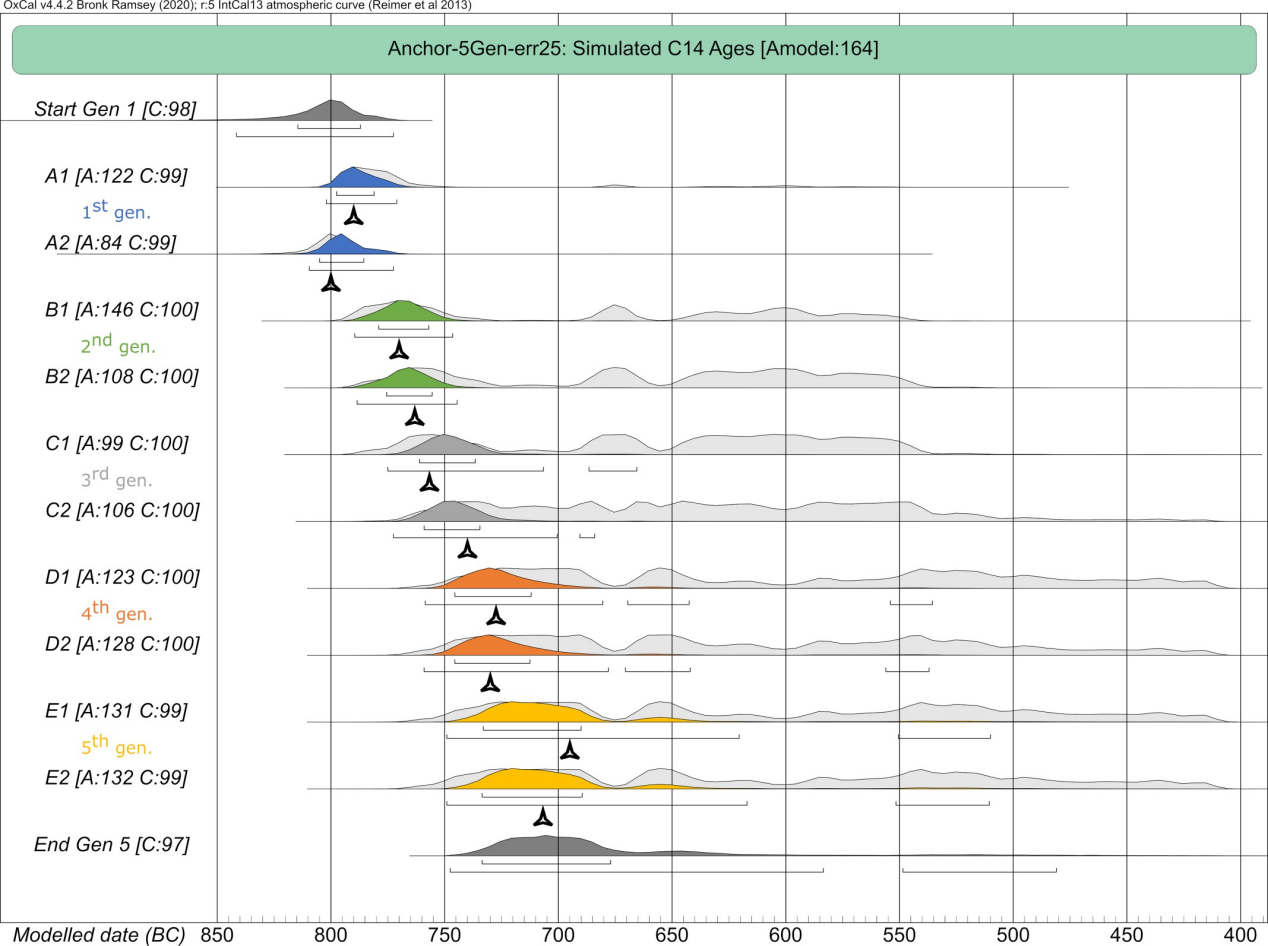
Plot of the five-generation Bayesian simulation with unmodelled and modelled dating range probabilities. (light grey) unmodelled probabilities; (coloured) modelled probabilities. Brackets underneath the probability ranges represent 2-sigma ranges of the modelled dates. Black triangles represent given/prescribed years of death (YoD).

All predefined or true YoDs lie within the modelled time frames. Simply sequencing the radiocarbon dates according to their generation level, without specific order within a generation, gives surprisingly good results, with an agreement index of the entire model of 164 and no value of single events lower than 97 (Fig 17). The agreement index gives a good estimate of how well any posterior distribution agrees with the prior distribution (model set up). The threshold for accepting the agreement is about 60% [39]. It also shows small probability areas of modelled dates from individuals D1-E2 after 600 cal BC, which are responsible for the overall wide ranges. The largest probability densities always incorporate the given YoDs.

Because of its special setting, improvements between unmodelled and modelled radiocarbon dates vary significantly (Fig 15 Panel III). The unmodelled dating range of A2 was already very small; therefore, the improvements are relatively restricted. In the five-generation model, they show 12 years of shortening, whereas those of C1-E2 range between 83 and 207 years. Improvements of dates from individual A1 are consistently high for models with 2–5 generations: up to 186 years. The modelled five-generation date of, e.g., B2 can now be used in archaeology for very fine dating, almost tightening possibilities down to one or two generations.

**Fig 18:**
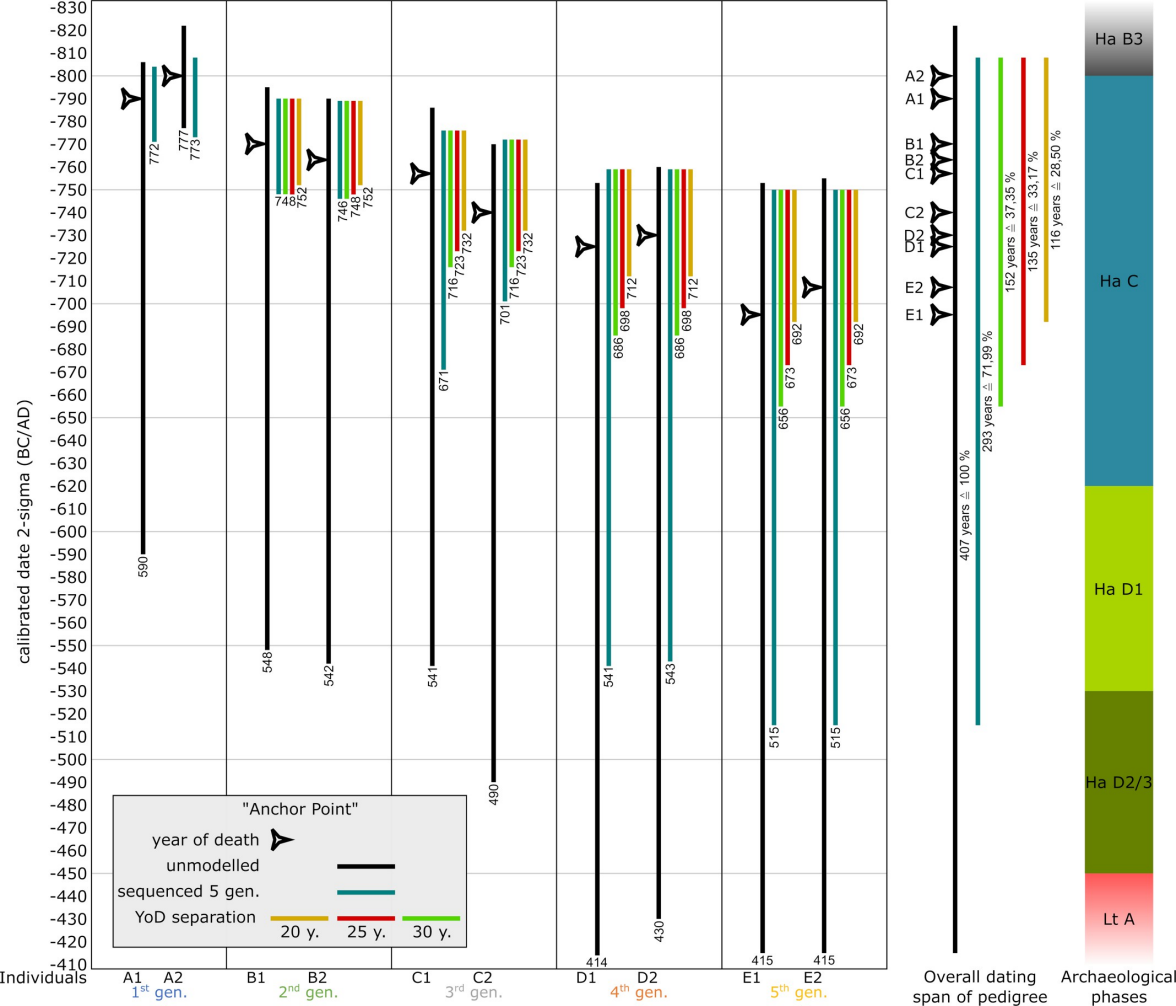
Combining pedigree-based Bayesian modelling and additional constraints. Unmodelled, calibrated date ranges (black) plotted against modelled ranges of simulation runs with 5 generations (blue) of the anchor point simulation. Additional constraints are results of varying YoD separations between individuals from subsequent generations (yellow: 20 years, red: 25 years and green: 30 years). Numbers below vertical bars are endpoints of dating ranges. Triangle symbols depict the actual YoD’s as inserted at the outset of the modelling process. The archaeological phases are based on the relative chronology of southern Germany.

In addition to the constraints solely based on Bayesian modelling, a YoD separation between individuals of two subsequent generations gives further substantial information on how to narrow down modelled dating ranges (following the basic guidelines of Sedig et al. 2021 [15]: there, YoD is called dates of death [DOD]). With more than two generations, those YoD-separations can be added up according to the number of generations. The value of assumed YoD-separation can be varied, resulting in slightly different outcomes of the entire pedigree. For demonstration purposes, we used three different YoD-separations: 20, 25 and 30 years, the latter reflecting the values supposed by Sedig et al. 2021 (28.84 years for parent-offspring relations) [15]. We also chose the anchor point simulation with five generations and the simple sequence setup to exemplify the power of those constraints in combination with the Bayesian approach (Fig 18).

Using only unmodelled dates, the entire dating span for the pedigree (potential duration) is 407 years (822–414 cal BC, 2-sigma), which is definitely too long for five generations of a family from pre-industrial times. Regarding archaeological dating, this would include the end of the Late Bronze Age until the beginning of the Latène period. Simply using the generation sequence in the Bayesian model, the entire span is reduced to 296 years (811–515 cal BC, 2-sigma).

The overall constraints of the entire pedigree are defined by the number of generations: the end of the 2-sigma-range from the latest individual of the 1^st^ generation + (n_gen_-1)*YoD separation in years. In our five-generation model, this would be the youngest end of the modelled 2-sigma-range of A1 and/or A2 + (5–1)*20/25/25 years to constrain the modelled dates of E1 and E2 and therefore the overall duration of the pedigree. Using a 20-year YoD separation results in -772 + 80 years = -692 or 692 cal BC; with a 30 year YoD-separation: 656 cal BC, because of the gap between the first and second generation, which is smaller than 30. The overall duration of the pedigree in the unmodelled version is 408 years, 296 years when sequenced and between 116 and 152 years when sequenced with varying YoD separations.

Looking at the details of the constraints for individuals of the last generation, the given/prescribed YoD of E1 = 695 and E2 = 707. Unmodelled 2-sigma ranges are E1 = 753–415 cal BC and E2 = 755–415 cal BC. Modelled 2-sigma ranges forE1 and E2 = 750–515 cal BC. Modelled 2-sigma ranges with additional YoD separation of 20 years give dating spans from 750 to 692 BC (58 years instead of 338/340 years). The improvement of dates is around 280/282 years when modelled and constrained. Even with this narrow YoD separation of 20 years, the prescribed YoDs fall within the widely constrained ranges. However, with an assumed generation gap of 25 or 29 years (for the latter, see [15]), the actual YoD of individuals E1 and E2 also fall within the constrained dating range of the pedigree: 25 years = 750–673 BC and 29 years = 750–659 BC. When applying these additional constraints, the entire pedigree is most probably situated in the first phase of the Hallstatt Period (Ha C).

Constraints/cut offs of the older ends of dating ranges do not apply here, because the differences between individuals of two subsequent generations do not exceed 18 years and are therefore even below the low margin of 20. It would only have an effect on individuals A1 and A2 in the plateau-simulation and A1 and A2, as well as D1 and D2, in the mixed simulation.

### Case studies

The aim of our three case studies is to look at every pedigree that exemplifies one specific problem/opportunity this biological information gives us to set up a Bayesian model, rather than looking at every possible prior or variable. The pedigree of POST, with its two strands, was analyzed by only sequencing the radiocarbon dates of individuals on the right side of it, with only adults incorporated, so that no descendant died before (one of) their parents. Additionally, we modelled once with a generation-gap between individuals 44 and 50/35 and once without, making it a pedigree with 4 or 5 generations in total. Because of its incompleteness and specific structure, the pedigree of OBKR can be modelled by sequencing on a generation level to show the potential of this method using only the most basic setup. The 2-generation pedigree of AITI demonstrates the difficulties of children comprising the pedigree when they possibly died before their parents. Varying arrangements of the chronological order in which the individuals died will affect the model setup and outcome. The three case studies also cover two different sections of the calibration curve, similar to the mixed simulation setup described above, and therefore display different initial situations of pedigrees.

**Fig 19:**
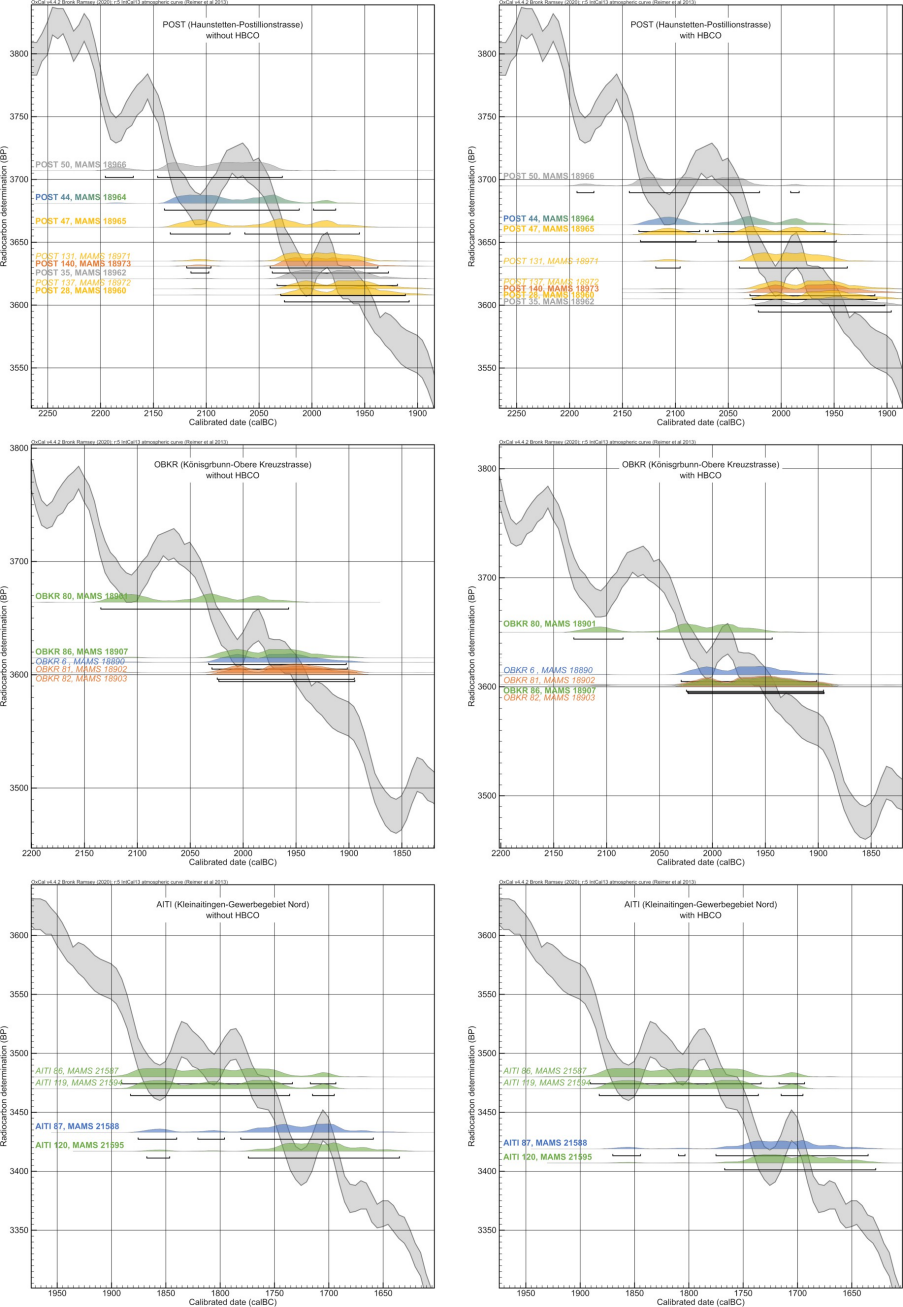
Comparison of radiocarbon dates without and with HBCO correction. Calibrated radiocarbon dates of individuals from the pedigrees plotted onto the calibration curve with 2-sigma probabilities and coloured according to their generation level. Left side: results of the radiocarbon measurements; right side: radiocarbon dates with HBCO correction. POST_44 is coloured with a blue to green fade because of its unclear assignment to either the 1^st^ or 2^nd^ generation (for results and parameters of the measurements and employed HBCO corrections according to Barta/Štolc 2007 [31], see. Table 1).

Incorporating AaD information through HBCO correction does not seem to make a big difference in the overall outcome of the calibrated, but unmodelled, dates (Fig 19). However, after incorporating HBCO correction into the Bayesian models of the three case studies, they predominantly produced lower agreement indices of the entire models and single measurements. Only the Bayesian model of OBKR benefited from HBCO correction (S5 Table). Adding an interval had no significant effect on the results of the modelling process. Therefore, all results discussed below are based solely on sequencing on a generation level, without HBCO correction or intervals. For demonstration purposes only, individually estimated intervals between individuals of two generations were incorporated in the five models of AITI. Bayesian models with HBCO correction and intervals in varying combinations, as well as additional setups (e.g., modelling the pedigree of POST with both strands), were calculated but are not discussed in detail here. Their OxCal codes and modelling results are integrated in the supplementary material (S6, S7 and S8 Tables and S5, S6 and S7 Codes).

#### Haunstetten—Postillionstraße (POST)

The overall dating range of the pedigree, affected by two subsequent wiggles on the calibration curve, spans 287 years (2196–1909 cal BC). The individual 2-sigma spans range from 114–188 years. In this case, we discarded the subadult individuals of the last generation (POST_131 and POST_137) placed on one strand of the pedigree because of the information gap in the 4^th^ generation and the uncertain placement of POST_137. Basically, two models were set up: the first one comprises four generations, with POST_44 being the uncle of POST_50, and the second one puts 44 as the grandfather of 50, adding another generation and resulting in a generation gap between them.

Based on the AaD estimates the following assumptions are made for the chronological order of deaths of the individuals: all individuals on the right side of the pedigree (Fig 7) were of adolescent age and most likely died in the order in accordance with their positioning in the pedigree. For example, even though it is possible that POST_140 died shortly before or at the same time as his mother (POST_35), the very similar unmodelled radiocarbon dates of both would lead to highly comparable results of constraints.

**Fig 20:**
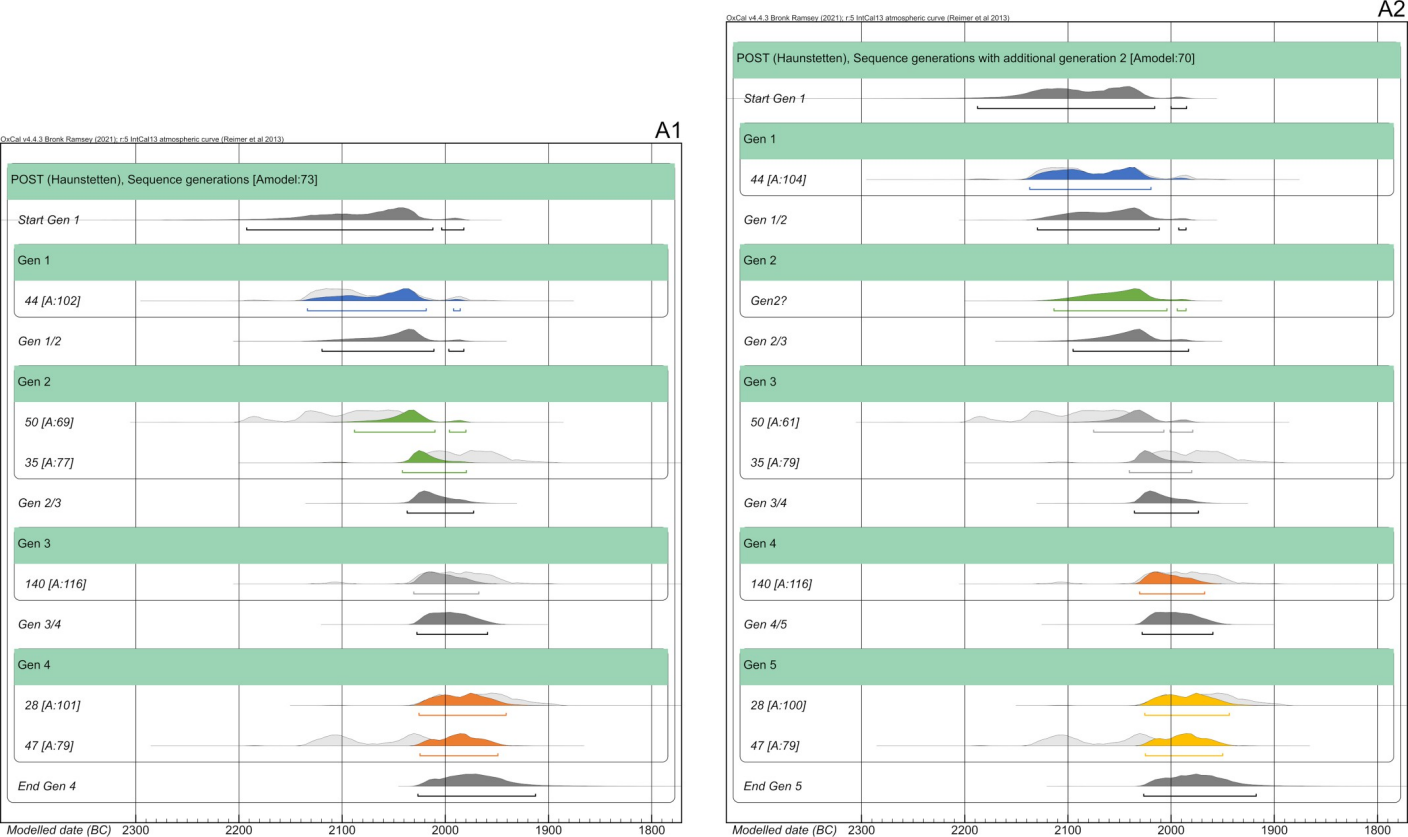
Bayesian modelling of the right strand of the pedigree of POST from sequencing the generations. (light grey) unmodelled probabilities; (coloured) modelled probabilities. (A1) Model with individual POST_44 being the uncle of POST_50. (A2) Model with POST_44 being the grandfather of POST_50 (see S6 Table Sheet 5 and 9).

Sequencing the pedigree with individual 44 being the uncle of 50, resulting in four generations in total, and not adding any other priors (HBCO, intervals) gives a satisfying outcome with an agreement index of the model of 73 and those of the single radiocarbon dates ranging between 69 and 116 (Fig 20A1). The shift of probability densities is most visible at individuals 50 and 47. Also, the oldest individual (POST_44) shows a shift towards the younger side. While the modelled dating range of 44 has not been constrained significantly (14 years), others did improve enormously (e.g., POST_140: 124 years, equaling 66.4%) (cf. Fig 25A1). The dating range of POST_35 was even constrained down to 62 years. From the oldest end of the dating range of generation 1 to the youngest end of generation 4, the pedigree now only covers 192 years, an improvement of 95 years in total.

Adding another generation between individuals 44 and 50 does not change the outcome of the entire pedigree significantly, and the agreement indices of the model, as well as single radiocarbon dates, do not show any differences (Figs 20A2 and 25A2). Unfortunately, in this case, the agreement indices do not yield any information about the positioning of individual 44 within the pedigree. The largest difference is the shortening of the younger end of the oldest individual 44, due to the subsequent generation.

#### Königsbrunn—Obere Kreuzstraße (OBKR)

In total, the overall dating range of the pedigree is 240 years (2135–1895 cal BC). The single results of the radiocarbon dating vary from 126 to 175 years (2-sigma). This pedigree is suitable for showing the simplest way of applying a Bayesian approach. The individuals and generations are directly put into chronological order and sequenced without further information of generation gaps, etc. Additional information about the gap in the 3^rd^ generation is ignored in this setup.

**Fig 21:**
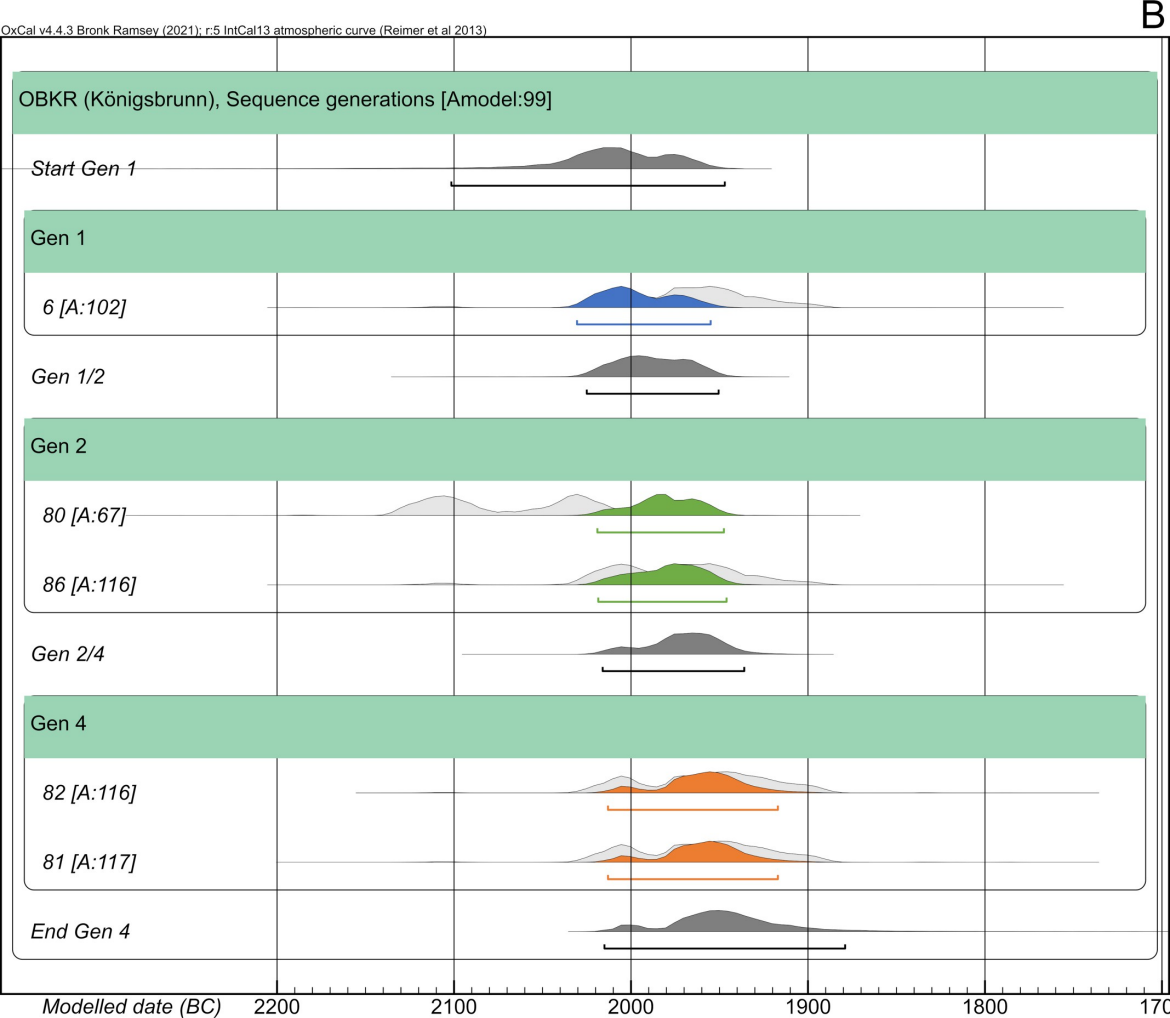
Bayesian modelling of the pedigree of OBKR. (light grey) unmodelled probabilities; (coloured) modelled probabilities. The process was run by directly sequencing the generations and ignoring the gap in the 3^rd^ generation (see S7 Table Sheet 1).

Overall, the agreement index of the model is surprisingly high (99), given the simple setup of the Bayesian model. Also, the single agreement indices range between 67 and 117, with four out of five lying above 100 (Fig 21). The radiocarbon dates of the last generation were constrained by 25%, resulting in a modelled time span of under 100 years (cf. Fig 25B). In contrast, the wide range of OBKR_80 was shortened at least by 105 years, an improvement of 59.3%, restricting the possible dating to the wiggle-section of the calibration curve between 2140 and 2040 cal BC. Because of the chronological order in comparison to his predecessor, OBKR_6 (unmodelled 2-sigma: 2030–1902 cal BC), who died at a young age of about 15–18 years, must have died before OBKR_80 (AaD: 30–40 years). In total, the entire timespan of the pedigree was shortened by 127 years, down to 113 years (2031–1918 cal BC), an improvement of 52.9%. As a result of this, each generation died at most roughly 28 years after the previous one.

#### Kleinaitingen—Gewerbegebiet Nord (AITI)

**Fig 22:**
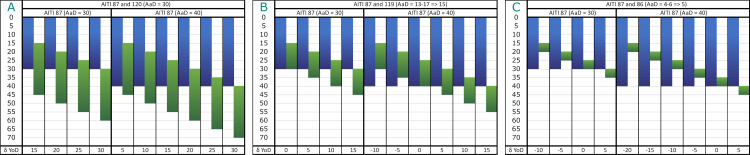
Compilation of scenarios of overlapping lifespans of the mother and her offspring. The possible lifespan of the mother (AITI_87) is depicted in blue, those of her sons (AITI_86, AITI_119 and AITI_120) are depicted in green. (A) mother and adolescent son AITI_120. (B) mother and subadult son AITI_119. (C) mother and subadult son AITI_86. δ YoD varies depending on the AaD estimations of the mother (30–40 years).

The overall dating span of this two-generation pedigree is 256 years (1891–1635 cal BC). However, this long dating range is caused by the prolonged wiggle of the calibration curve between 1880 and 1760 cal BC. We will demonstrate the possibilities and challenges of pedigree-based Bayesian modelling when parents had children that eventually died before them. Therefore, this pedigree was broken down into three pairwise Bayesian models. The main issue here is the chronological order of times of death (Fig 22). The first pair (Fig 22A), mother (AITI_87) and son (AITI_120), contains two adult individuals, and the chronological order of YoDs follows the structure of the pedigree. Depending on the AaD of the mother, her son AITI_119 could have died earlier, simultaneously or later (Fig 22B). Her son AITI_86 died at a very young age, most likely before her (Fig 22C). The calculation of intervals is dependent on the chronological order of deaths and covers the δ YoD timespan.

In total, five different specifications of the Bayesian model have been calculated, which are the result of the chronological order of YoDs:

1. AITI_87 died before or simultaneously with AITI_120, with an interval of 20 ± 10 years.
2. AITI_87 died before or simultaneously with AITI_119, with an interval of 7.5 ± 7.5 years.
3. AITI_119 died before or simultaneously with AITI_87, with an interval of 5 ± 5 years.
4. AITI_87 died before or simultaneously with AITI_86, with an interval of 2.5 ± 2.5 years.
5. AITI_86 died before or simultaneously with AITI_87, with an interval of 10 ± 10 years.

Just by looking at the order of unmodelled radiocarbon dates plotted against the calibration curve, the above indicated orders of YoDs seemed to be verified already, where the mother died after her two sons AITI_86 and AITI_119, whereas the adult son AITI_120 died after her.

**Fig 23:**
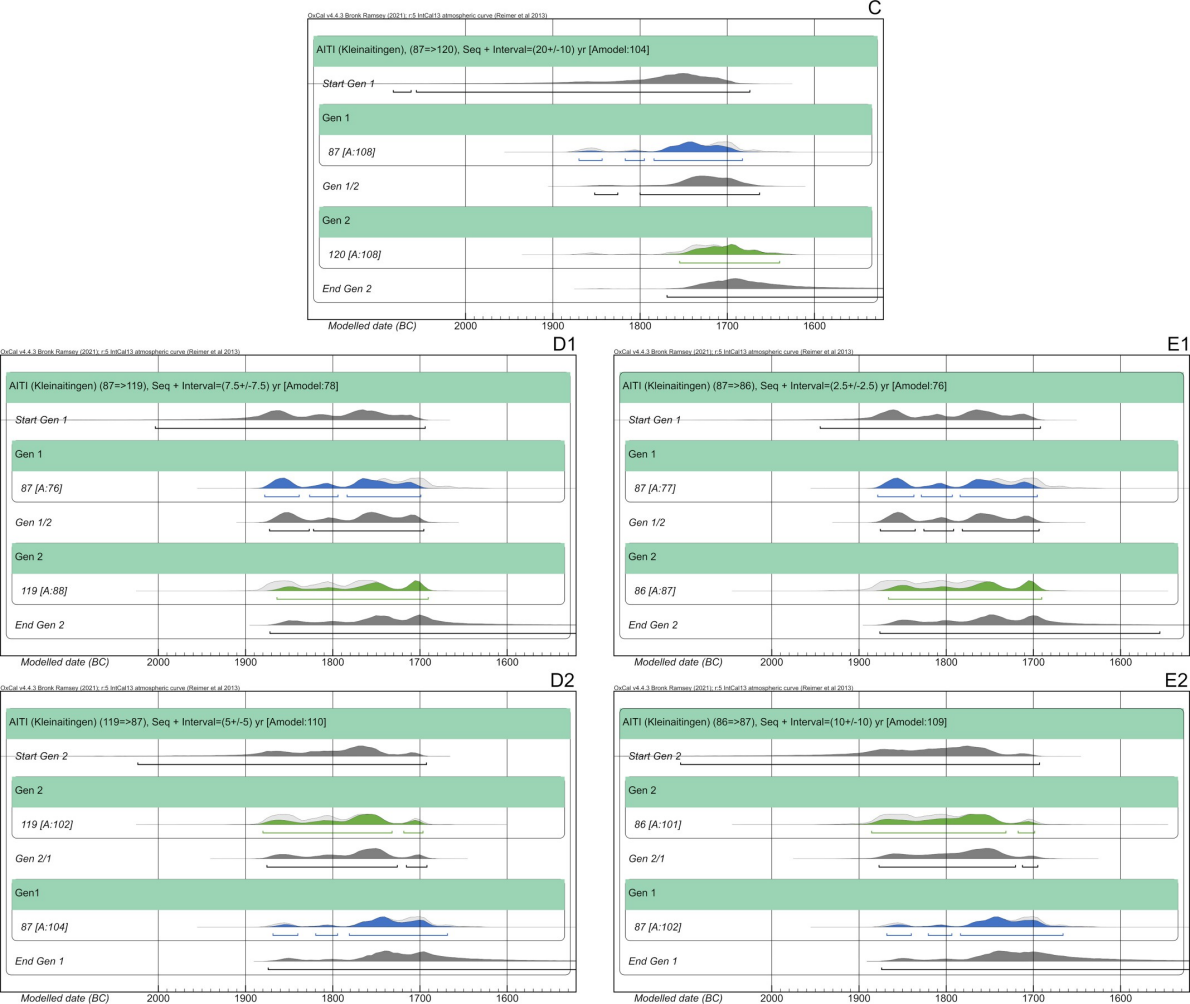
Results of pairwise Bayesian modelling of the mother (AITI_87) and her sons. (light grey) unmodelled probabilities; (coloured) modelled probabilities. The switched chronological order of AITI_87 and AITI_119 (D1 and D2), as well as AITI_86 (E1 and E2), are plotted separately (see S8 Table Sheet 7, 11, 15, 19 and 23).

The Bayesian model including a 20 ± 10 year interval between mother (AITI_87) and adult son (AITI_120) works quite well and shows an agreement index of the model of 104; the single radiocarbon dates are comparable (108) (Fig 23C). While the modelled dating range of the mother only shows a low improvement, the sons’ has been constrained by approximately half (cf. Fig 25C).

Modelling the other two pairs (AITI_87 with AITI_119 and AITI_86) does not result in a significant reduction of the modelled date ranges in comparison with the unmodelled ones, no matter in which chronological order the generations are placed (cf. Fig 25D1–25E2). Nevertheless, the differences in agreement indices between the models where the mother died first (78 and 76: Fig 23D1 and 23E1) and where the two sons died earlier (110 and 109: Fig 23D2 and 23E2) are compelling indicators that the mother survived her sons AITI_86 and AITI_119.

**Fig 24:**
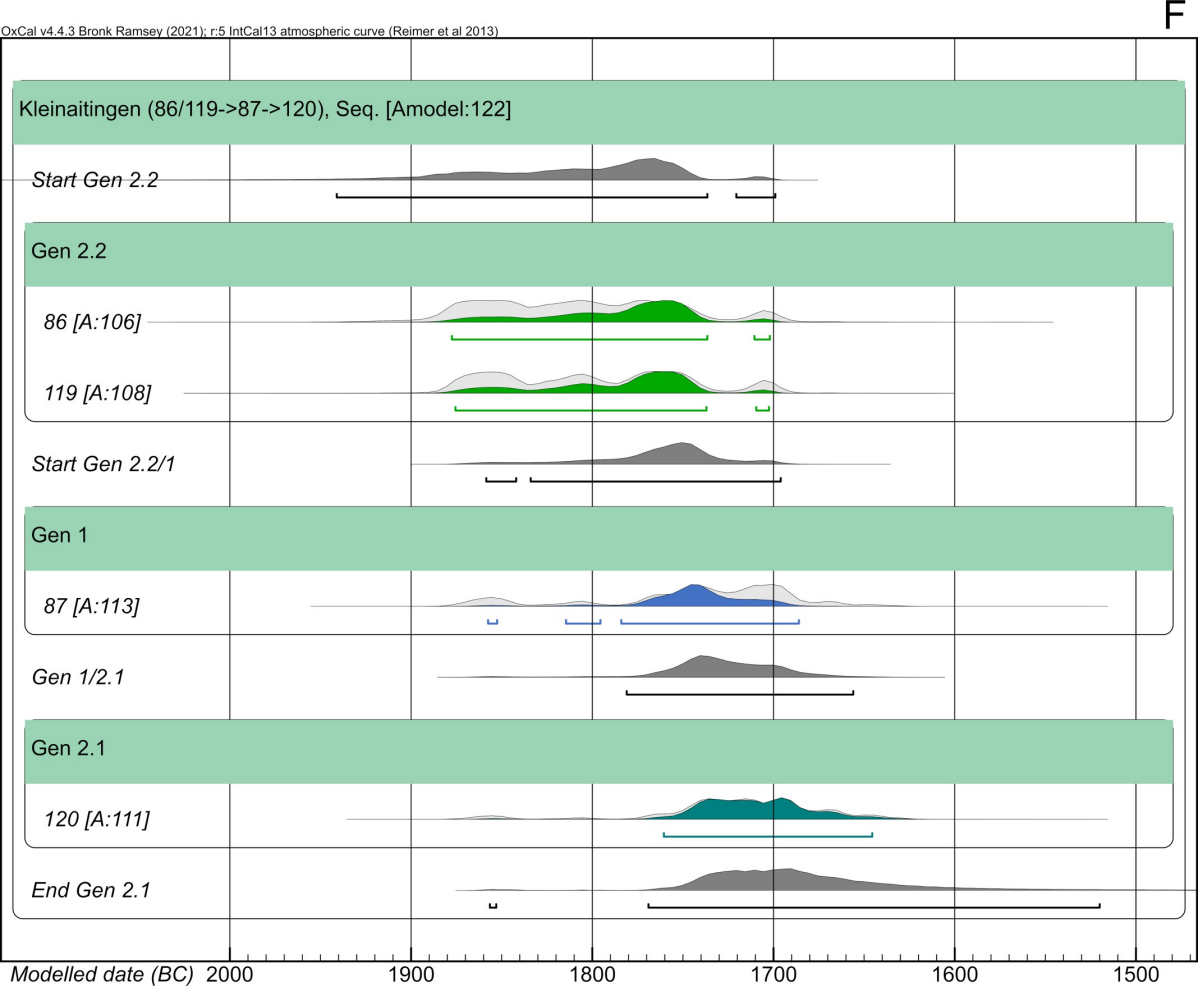
Results of Bayesian modelling of the entire pedigree of AITI. The process was run by directly sequencing the individuals in their proposed chronological order of death without intervals in between (see S8 Table Sheet 25).

**Fig 25:**
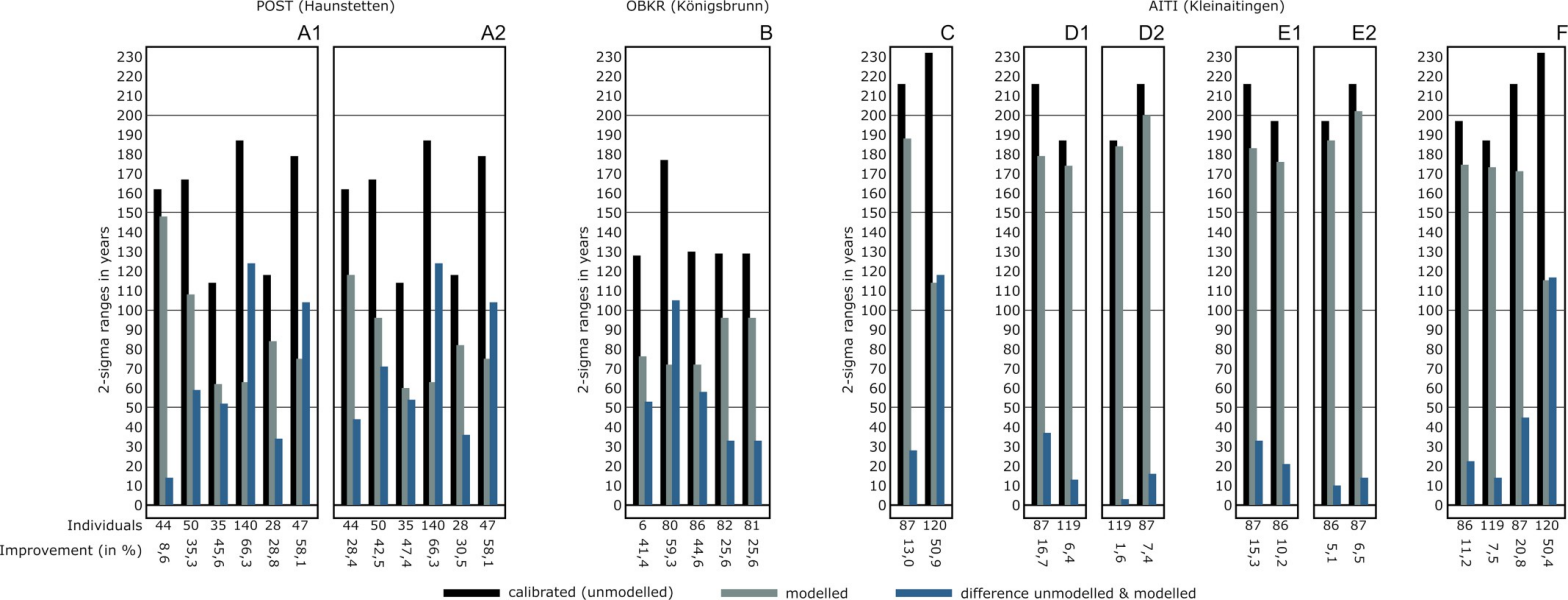
Compilation of the results from Bayesian modelling of the case studies. Unmodelled (black) and modelled (grey) dating ranges (2-sigma) and their resulting improvements (blue) are shown for each case mentioned before (see Figs 20, 21, 23 and 24).

Moreover, the probability densities of AITI_87 in models C, D2 and E2 are surprisingly similar, which is yet another indicator that AITI_119 and AITI_86 died before their mother AITI_87 and brother AITI_120 (see. S5 Table). It is therefore not an inversion of unmodelled dates as seen in the Hallstatt plateau simulation. The results from the pairwise modelling of the mother with each child of hers can finally be used to set up a Bayesian model for the entire family (Fig 24). All agreement indices are above 100; the model even shows an agreement of 122. Therefore, the proposed sequence of deaths is very likely. Unfortunately, as seen in the pairwise model setups, the constraints of the single radiocarbon dates have not improved considerably (Fig 25F). The overall dating range of the pedigree has only dropped by 24 years (1878–1646 cal BC). However, because of the probability density of the modelled date range from AITI_87, it seems very likely that the dating ranges of AITI_86 and AITI_119 can be constrained to a beginning around 1800 cal BC, following the method of J. Sedig and colleagues [15].

## Conclusions and outlook

One of the major results of the four simulations using artificial pedigrees is that the sequencing of individuals grouped in generations without additional priors already has a substantial effect on the constraints, whereby Bayesian modelling is applied to the radiocarbon dates based upon information of biological relationships.

Recognisable improvements to the precision of calibrated dates were achieved at all three main layouts of the calibration curve (plateau, steep, mixed). Both simulations placed within the Hallstatt plateau (plateau and anchor point) showed that Bayesian modelling excluded a dating in the second half of the Hallstatt plateau (after 530 cal BC), an enhancement with high relevance for an archaeological chrono-interpretation. Basically, our method is applicable to pedigrees at all sections of the calibration curve and therefore for all detected family trees of the past with sufficient coverage of radiocarbon dates. Constraints that impact the modelling results are the precision of the single radiocarbon dates, the number of modelled generations, the shape of the calibration curve and the availability of “anchor points”.

Integrating additional priors can lead to slightly different outcomes: adding an interval between generations can have a positive or negative effect on the precision of the calibrated ages in comparison to sequence-only simulations due to the specific shape of the calibration curve and the exact chronological positioning of the individuals of the pedigree. Adding intergenerational intervals did not change the results at the steep and mixed simulations significantly. In the plateau simulation, it reduced the modelled timespans, while they increased for the anchor point simulation.

One could also use further pedigree information if available, e. g. caused by a larger number of individuals within a generation, to prescribe a length to a generation [“Span()”] within the OxCal code (similar shown in Meadows et al. 2020 [5]), which may also help to constrain calibrated date-ranges.

The bracketing effect always reduces modelled dating ranges, at least for the first run. Adding yet another subsequent generation level to the pedigree did not further improve those in the steep and mixed simulation. In the plateau simulation, this continuous improvement was only visible in the setup with intervals, whereas the modelled radiocarbon dates got constrained bit by bit with each generation level added in the anchor point simulation, which can be attributed to the role of the “anchor point” of the pedigree and the consequences thereof.

Supposed inversions of radiocarbon dates, which are always a cause of the specific shape of the calibration curve, can be partly or almost entirely resolved through Bayesian modelling based upon information from the pedigree.

Adding further constraints after modelling based on duration estimates of generation levels and entire pedigrees (see e.g. [15]) can even improve the outcome of the models significantly. In our anchor point simulation, these constraints led to a highly reduced overall dating span of the pedigree, which stretches only 28–37% of the time range compared to the unmodelled single radiocarbon dates.

Similar to our anchor point simulation, dendro dates of graves with a very narrow dating range can be used as an anchor point for the Bayesian model (c.f. Early Bronze Age princely burials of Helmsdorf and Leubingen, Germany; [40,41]). Especially in periods with a flat calibration curve (e.g., Hallstatt plateau), those chronological anchor points can lead to a substantial improvement of radiocarbon dates from individuals who are biologically related to those who have been dated with dendrochronology (e.g., the Hallstatt period site of Magdalenenberg in Baden-Württemberg, Germany; [42]).

The case studies presented in this paper partly demonstrate the complexity of real pedigrees but also their incompleteness. Every site and pedigree provides a varying amount and quality of information, which can be used for a Bayesian model of the radiocarbon dates (c.f. [5]). Therefore, setting up a Bayesian model for a real pedigree is always afflicted with information gaps and site-specific problems but also opportunities.

In our first case study of POST, we demonstrated the possibility to divide a multi-strand pedigree into separate parts. For one part, we applied our Bayesian approach, because of its high density of information. Here, the most probable chronological order of deaths of the individuals was most important, as they all died as adults and an inversion of YoDs compared to their generation level is unlikely.

The power of a simple Bayesian model setup was demonstrated in the pedigree of OBKR, where no additional priors were included and an information gap in the 3^rd^ generation was also neglected. Nevertheless, the model proved its applicability by resulting in a calculated overall duration of 113 years for four generations instead of 240 years and a major improvement of single radiocarbon dates for individuals from within the pedigree.

In contrast to the significant constraints of radiocarbon dates in the other two case studies, the Bayesian model of the pedigree of AITI has proven yet another application of this method. Here, the chronological order of the YoDs of the mother and her three children was unknown before modelling. The sequence of deaths within this nuclear family was plausibly reconstructed through pairwise comparisons in individual Bayesian models (see Fig 24). Those pairwise comparisons can be implemented in every other Bayesian model of a pedigree where children possibly died before their parents, therefore inverting the presumed generation-based chronological order. But we are also aware that not every chronological order between individuals of two generations can be resolved in the same way as shown in the case of AITI. This is mainly based on the shape of the calibration curve and the quality of information the pedigree and its radiocarbon dates provide.

A seeming inversion of unmodelled radiocarbon dates is seen in all three case studies. Without any stratigraphic information, resolving these is only possible with Bayesian modelling and prior information. Our suggested simulations are of course only an approximation of the reality; nonetheless, the priors and parameters can be adapted and complemented according to the specific research questions and contexts. Hereby, we chose a setup without chronologically overlapping generations and only adult individuals in a single-stranded pedigree. In most cases, real family trees could be way more complex concerning structure, layout and information gaps. Therefore, we suggest not to simply copy the OxCal code for upcoming studies without carefully checking its applicability in specific cases.

Our case studies show that using pedigree information does actually work when sequencing only on a generation level without priors and does often improve calibrated ages significantly. In general, applying a Bayesian approach to radiocarbon dates based on biological relations is and will be of major importance at improving dating accuracy in future studies.

Our simulations were run on single-strand pedigrees, but radiocarbon dated multi-strand pedigrees (S2 Fig) can be modelled using our method as well, even if each strand should be calculated separately. This is most important to evaluate when modelling pedigrees with several individuals in a generation level, for example multiple siblings or cousins. Especially pedigrees with multiple generation levels and more than one strand will have to be carefully evaluated before a Bayesian model and the needed priors is set up to prevent inconsistencies for example caused by overlapping generations. Information gaps—missing or imprecise radiocarbon dates, as well as missing aDNA data—are of minor significance to the outcome of the modelling process, but refined priors and variables could help to reach higher levels of constraints for the radiocarbon dates.

Upcoming studies in different fields of research will deliver evidence for some of the parameters/priors used to set a Bayesian model, like higher accuracy in determining the AaD (e.g. [43]), the age at which particular females gave birth and so forth. All these parameters are modifiable in the set of models provided in this paper. Ancient DNA analyses are already state of the art in many ongoing archaeological projects dealing with human, animal or even plant remains. Their significance, frequency and scientific power will certainly increase dramatically in the years and decades to come. Therefore, reconstructing biological pedigrees of humans or even animals will probably become a standard practice and give us the chance to apply the presented methods in this paper to a very large extent. This is also true for times in which the flat calibration curve hinders us from working with unmodelled dates, such as the Hallstatt plateau.

## Supporting information

S1 Fig Compilation of radiocarbon dates from 6600–2010 BP with varying uncertainties. Lower panel: calibrated radiocarbon dates (2-sigma) with 10 (black), 20 (grey) and 30 (red) years uncertainty in 5-year intervals from approximately 5600 cal BC to 25 cal AD. Upper panel: differences of the 2-sigma ranges between 10 and 30 years of uncertainty.
(TIF)

S2 Fig Artificial multi-strand pedigree with five generations. Each branch of the pedigree can be modelled separately or put into a Bayesian model including every individual and/or strand.
(TIF)

S3 Fig Age-at-death estimations (AaD) in years for the individuals used within the case studies. (green) AaD minima; (blue) AaD uncertainty added to AaD min. Overall average of AaD uncertainties is 8.06 years. All parameters of the case studies are listed in Table 1.
(TIF)

S1 Table Outputs and raw data of the Bayesian modelling of the plateau model. The file contains four sheets, each one with an added generation level (summarized results in Fig 5, OxCal Codes provided in S1 Codes).
(XLSX)

S2 Table Outputs and raw data of the Bayesian modelling of the steep model. The file contains four sheets, each one with an added generation level (summarized results in Fig 5, OxCal Codes provided in S2 Codes).
(XLSX)

S3 Table Outputs and raw data of the Bayesian modelling of the mixed model. The file contains four sheets, each one with an added generation level (summarized results in Fig 5, OxCal Codes provided in S3 Codes).
(XLSX)

S4 Table Outputs and raw data of the Bayesian modelling of the anchor point model. The file contains four sheets, each one with an added generation level (summarized results in Fig 5, OxCal Codes provided in S4 Codes).
(XLSX)

S5 Table Agreement indices of the Bayesian models according to the different variables used in the case studies. Agreement indices for single radiocarbon dates as well as for the entire model above 60% are considered to show the accordance between the model and the measured dating probabilities.
(XLSX)

S6 Table Outputs and raw data of the Bayesian modelling of the pedigree of POST. In each sheet (1–12) a varying bayesian model was calculated using different variables (HBCO and intervals). (1–4) entire pedigree with four generations (POST_44 being the uncle of POST_50). (5–8) only the right strand of the four generation pedigree with adult individuals. (9–12) only the right strand of the five generation pedigree with adult individuals (POST_44 being the grandfather of POST_50) (OxCal Codes provided in S5 Codes).
(XLSX)

S7 Table Outputs and raw data of the Bayesian modelling of the pedigree of OBKR. In each sheet (1–4) a varying Bayesian model was calculated using different variables (HBCO and intervals) (OxCal Codes provided in S6 Codes).
(XLSX)

S8 Table Outputs and raw data of the Bayesian modelling of the pedigree of AITI. In each sheet (1–25) a varying bayesian model was calculated using different variables (HBCO and intervals). (1–4) entire pedigree with two generations, with the sequence in the Bayesian model according to their generation level (OxCal Codes provided in S7 Codes). (5–24) pairwise Bayesian modelling (mother together with one of her sons) (OxCal Codes provided in S8, S9, S10, S11 and S12 Codes). (25) entire pedigree with the individuals assigned to sequence levels based on the results of the pairwise comparisons (5–24) (OxCal Codes provided in S13 Codes).
(XLSX)

S1 Text Anthropological information. General remarks and supporting information about the case studies.
(DOCX)

S1 Codes OxCal codes for the Bayesian modelling of the plateau model (results in S1 Table). (LOG)

S2 Codes OxCal codes for the Bayesian modelling of the steep model (results in S2 Table). (LOG)

S3 Codes OxCal codes for the Bayesian modelling of the mixed model (results in S3 Table). (LOG)

S4 Codes OxCal codes for the Bayesian modelling of the anchor point model (results in S4 Table). (TXT)

S5 Codes OxCal codes for the Bayesian modelling of the pedigree of POST. The codes are labeled according to the sheets in S6 Table (1–12).
(TXT)

S6 Codes OxCal codes for the Bayesian modelling of the pedigree of OBKR. The codes are labeled according to the sheets in S7 Table (1–4).
(TXT)

S7 Codes OxCal codes for the Bayesian modelling of the entire pedigree of AITI. The codes are labeled according to the sheets in S8 Table (1–4). The interval (25 ± 10 years) is based on observations in Fig 22.
(TXT)

S8 Codes OxCal codes for the Bayesian modelling of the individual AITI_87 followed by AITI_120. The codes are labeled according to the sheets in S8 Table (5–8) The interval (20 ± 10 years) is based on observations in Fig 22.
(TXT)

S9 Codes OxCal codes for the Bayesian modelling of the individual AITI_87 followed by AITI_119. The codes are labeled according to the sheets in S8 Table (9–12) The interval (7.5 ± 7.5 years) is based on observations in Fig 22.
(TXT)

S10 Codes OxCal codes for the Bayesian modelling of the individual AITI_119 followed by AITI_87. The codes are labeled according to the sheets in S8 Table (13–16) The interval (5 ± 5 years) is based on observations in Fig 22.
(TXT)

S11 Codes OxCal codes for the Bayesian modelling of the individual AITI_87 followed by AITI_86. The codes are labeled according to the sheets in S8 Table (17–20) The interval (2.5 ± 2.5 years) is based on observations in Fig 22.
(TXT)

S12 Codes OxCal codes for the Bayesian modelling of the individual AITI_86 followed by AITI_87. The codes are labeled according to the sheets in S8 Table (21–24) The interval (10 ± 10 years) is based on observations in Fig 22.
(TXT)

S13 Codes OxCal codes for the Bayesian modelling of the entire pedigree of AITI with changed chronological order. The individuals are assigned to sequence levels based on the results of the pairwise comparisons (cf. Fig 23) and modelled without HBCO or intervals. The code is labeled according to the sheets in S8 Table (25).
(TXT)
