# Supplementary figures and images for: Pedigree-based Bayesian modelling of radiocarbon dates
Source: PLoS One. 2022 Jun 30;17(6):e0270374. doi: 10.1371/journal.pone.0270374 (PMC9246184; doi:10.1371/journal.pone.0270374)

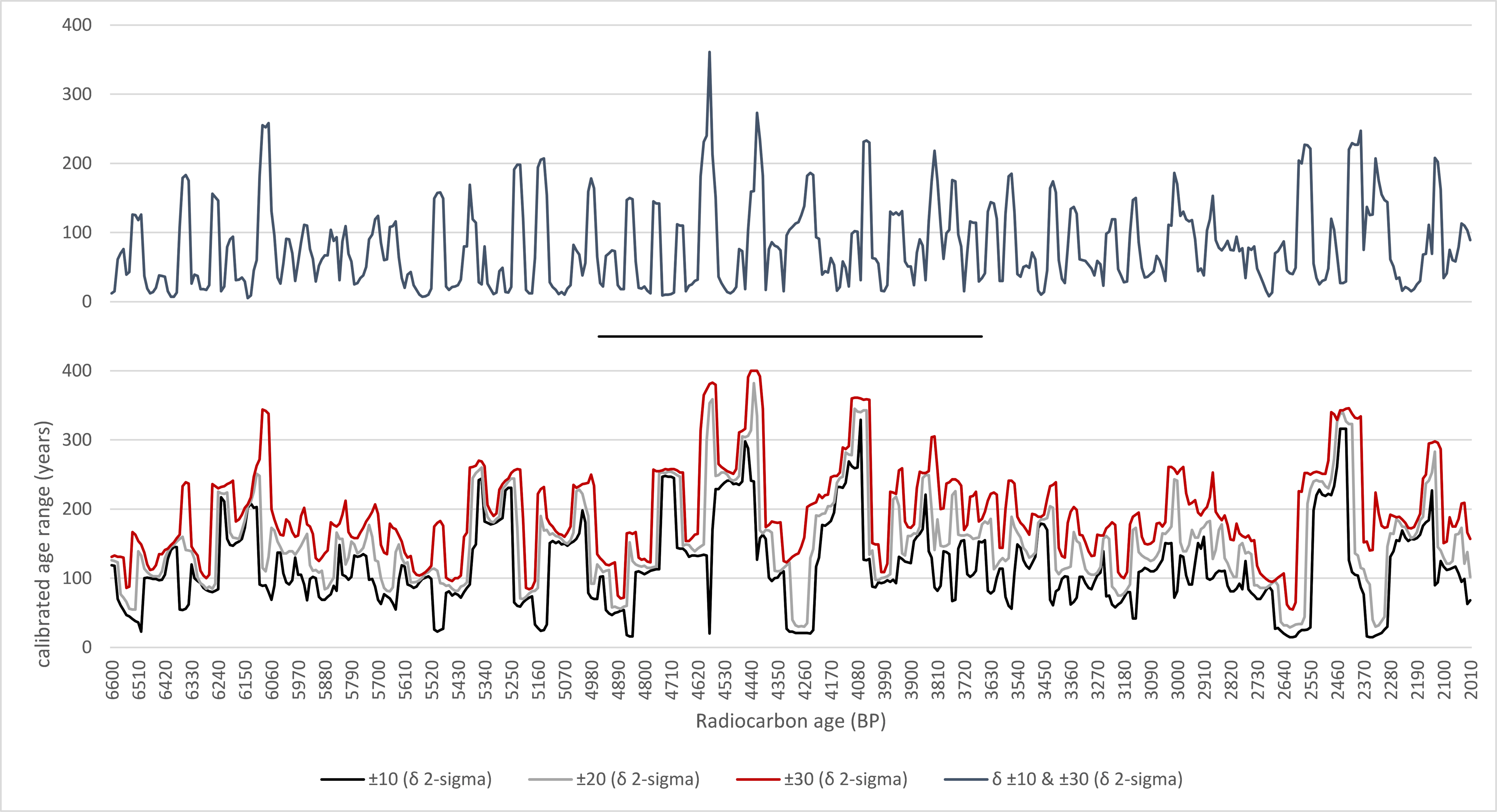

Supplement: S1 Fig — Lower panel: calibrated radiocarbon dates (2-sigma) with 10 (black), 20 (grey) and 30 (red) years uncertainty in 5-year intervals from approximately 5600 cal BC to 25 cal AD. Upper panel: differences of the 2-sigma ranges between 10 and 30 years of uncertainty. (TIF) [file pone.0270374.s001.tif]

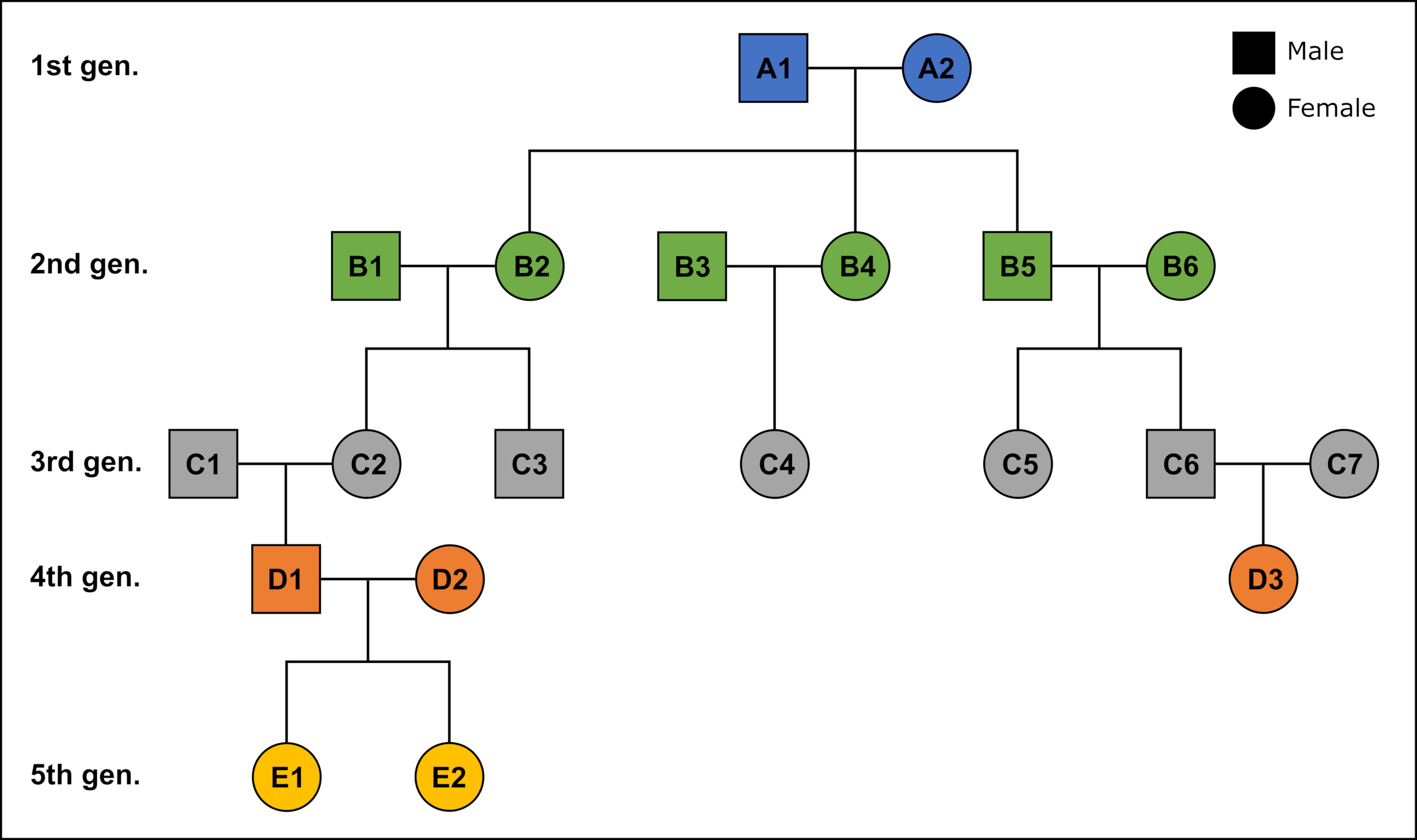

Supplement: S2 Fig — Each branch of the pedigree can be modelled separately or put into a Bayesian model including every individual and/or strand. (TIF) [file pone.0270374.s002.tif]

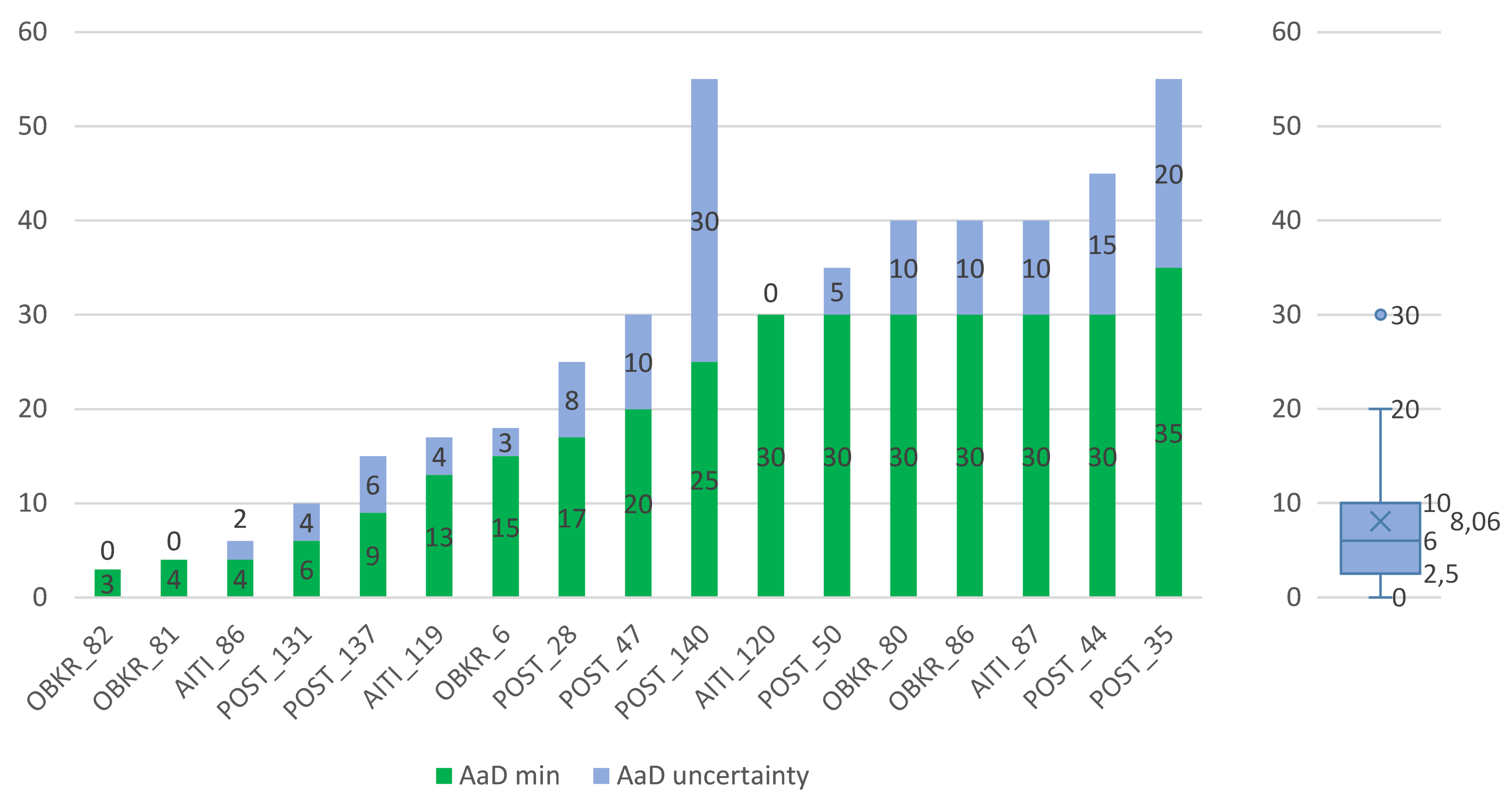

Supplement: S3 Fig — (green) AaD minima; (blue) AaD uncertainty added to AaD min. Overall average of AaD uncertainties is 8.06 years. All parameters of the case studies are listed in Table 1. (TIF) [file pone.0270374.s003.tif]
