# Supplementary material for: Pedigree-based Bayesian modelling of radiocarbon dates
Source: PLoS One. 2022 Jun 30;17(6):e0270374. doi: 10.1371/journal.pone.0270374 (PMC9246184; doi:10.1371/journal.pone.0270374)
Supplement: S1 Text — General remarks and supporting information about the case studies. (DOCX) [file pone.0270374.s012.docx]

Anthropological information

For the method used in this Bayesian approach, an anthropological AaD determination is indispensable in order to define the chronological order of YoDs of the individuals (e.g. children dying before parents) or to calculate the HBCO correction [30]. The precision of the AaD estimates depends on various parameters, such as the state of preservation of the bones, representative status of the skeletal remains and the age of the deceased at death. Often, soil conditions are key to the preservation of bone material. Acidic soil disintegrates or dissolves bones, and gravel causes physical damage which hampers the osteological analysis. The more diagnostic features on the skeletal remains for an AaD estimate of a single individual are available (e.g. cranial sutures, status of the epiphyseal plates, dental status, etc.), the more precise their estimates. Especially in archaeological contexts with successive burial activities and partial removal or deposition of skeletal parts, this is a limiting factor. In our case study, POST_140 was partially destroyed during construction work during the archaeological excavation, leaving only half of the skeletal remains intact and resulting in the large AaD estimate uncertainty of 30 years. In general, the AaD span is smaller in young individuals than in older ones, as fewer developmental osteological milestones are reached after adolescence. All individuals of the cemeteries used for the case studies were examined anthropologically, showing a normal demographic distribution for pre-industrial societies. Based on the morphological examinations of the skeletal remains from OBKR and AITI, approximately two thirds of all adult individuals died between the age of 20 and 40 years (OBKR: 65,2 %, AITI: 66,2 %), with most of them not surpassing 30 years of age [37]. Uncertainties of the AaD estimates from individuals in the reconstructed pedigrees of all three sites range from 0-30 years, with a low overall average of 8,06 years (S3 Fig). The sex assessment via morphological or aDNA analysis is only needed to reconstruct pedigrees.
